# Abiotic synthesis during the interaction of ferrous chloride–rich silicic fluids with marble under high-grade metamorphic conditions

**DOI:** 10.1073/pnas.2423043122

**Published:** 2025-08-28

**Authors:** Chenhui Fei, Shun Guo, Yibing Li, Jingbo Liu

**Affiliations:** ^a^State Key Laboratory of Lithospheric and Environmental Coevolution, Institute of Geology and Geophysics, Chinese Academy of Sciences, Beijing 100029, China; ^b^Marine Science and Technology College, Zhejiang Ocean University, Zhoushan 316022, China; ^c^College of Earth and Planetary Sciences, University of Chinese Academy of Sciences, Beijing 100049, China

**Keywords:** ferrous chloride–rich silicic fluids, fluid–marble interaction, organic species, abiotic organic synthesis, Sulu UHP metamorphic terrain

## Abstract

Ferrous chloride–rich silicic fluid and melt infiltration led to the decarbonation of dolomitic marble in the Chinese Sulu ultrahigh-pressure metamorphic terrain under temperatures ranging from 670 to 800 °C, pressures from the aragonite + albite to calcite stability fields and the oxygen fugacities between the hematite–magnetite and pyrite–pyrrhotite–magnetite buffers, resulting in the formation of olivine marble and diopsidite. The inclusions in zircons trapped during the decarbonation process suggest that the H_2_-producing reaction 3FeCl_2_ (aq) + 3CaCO_3_ + H_2_O = Fe_3_O_4_ (magnetite) + 3CaCl_2_ (aq) + 3CO_2_ + H_2_ occurred and that it induced magnetite-catalyzed Fischer–Tropsch-type synthesis, as indicated by the presence of whewellite, disordered carbonaceous material, CH_4_, and CO in the inclusions. The results of this study highlight the role of aqueous Fe in generating H_2_ and magnetite and have far-reaching implications for carbon speciation and solubility in deep fluids and for endogenic abiotic synthesis, which may be pivotal in the prebiotic occurrence of organic compounds on Earth.

## Significance

Abiotic organic synthesis in geological processes is closely linked to the Earth’s carbon cycle and the origin of early life. Our research revealed that the infiltration of ferrous chloride–rich silicic fluids into dolomitic marble induced a decarbonation reaction (T >670 °C, P >1.0 GPa) that produced H_2_ and magnetite, facilitating magnetite-catalyzed organic synthesis. This study highlights the role of aqueous Fe in the generation of H_2_ and magnetite and extends the scope of research on abiotic organic synthesis in natural samples from low-grade to high-grade metamorphic conditions.

The generation of H_2_ during fluid–rock interactions is considered to play a key role in the formation of abiotic organic compounds via Fischer–Tropsch-type (FTT) synthesis (1–5). The general H_2_-producing reaction is described as 2FeO (in minerals) + H_2_O → Fe_2_O_3_ (in minerals) + H_2_ (1–3). In this process, FTT synthesis can occur via the reduction of CO_2_, CO, or HCO_3_^−^ with H_2_ in the presence of some kind of mineral catalyst, such as magnetite (1, 6, 7), and this process has been used to explain the origin of condensed carbonaceous matter in serpentinized ultramafic rocks (8, 9) and the origin of aliphatic hydrocarbons in altered alkaline igneous rocks (10–13).

Iron, as one of the most abundant elements in the Earth’s crust, is also a major aqueous solute in a variety of hydrothermal circumstances (14). However, little attention has been paid to the role of aqueous Fe in the abiotic synthesis of organic compounds. Fe is highly soluble in Cl-bearing fluids, and Fe^2+^–Cl complexes are the predominant species within a wide range of oxygen fugacities buffered by hematite–magnetite (HM), pyrite–pyrrhotite–magnetite (PPM), and fayalite–magnetite–quartz (14–16). Fe^2+^ chloride fluids can react with calcite to produce skarn-type magnetite ore deposits and generate H_2_ and CO_2_ (15), producing a potential system for magnetite-catalyzed FTT synthesis.

In this study, we examined olivine marble and diopsidite present as lenses in stromatic migmatites from the Weihai region of the Sulu ultrahigh-pressure (UHP) metamorphic terrain, which were formed by decarbonation due to the interaction of dolomitic marble with infiltrating Fe^2+^ chloride–rich silicic fluids and partial melts. The inclusions in zircons from the two rocks have been studied, and the results reveal the role of aqueous Fe in the generation of H_2_ and magnetite and the presence of abiotic organic synthesis during the decarbonation process of marbles. In addition, the abiotic origin of organic compounds under low-temperature hydrothermal circumstances has recently been called into question since the hydrothermal fluids with surface waters can supply biotic organic matter for altered rocks (17). However, the rocks in this study formed under high-grade metamorphic conditions, where it is impossible for hydrothermal fluids to interact with surface waters. Even if there are biotic organic compounds in the protolith before metamorphism, they will be carbonized and graphitized during progressive metamorphism (18, 19). Therefore, any finding of indigenous organic materials in such high-grade metamorphic rocks is conclusive evidence for the occurrence of abiotic synthesis. In this study, mineral abbreviations follow ref. 20.

## Results

### Petrology.

**Fig. 1.**
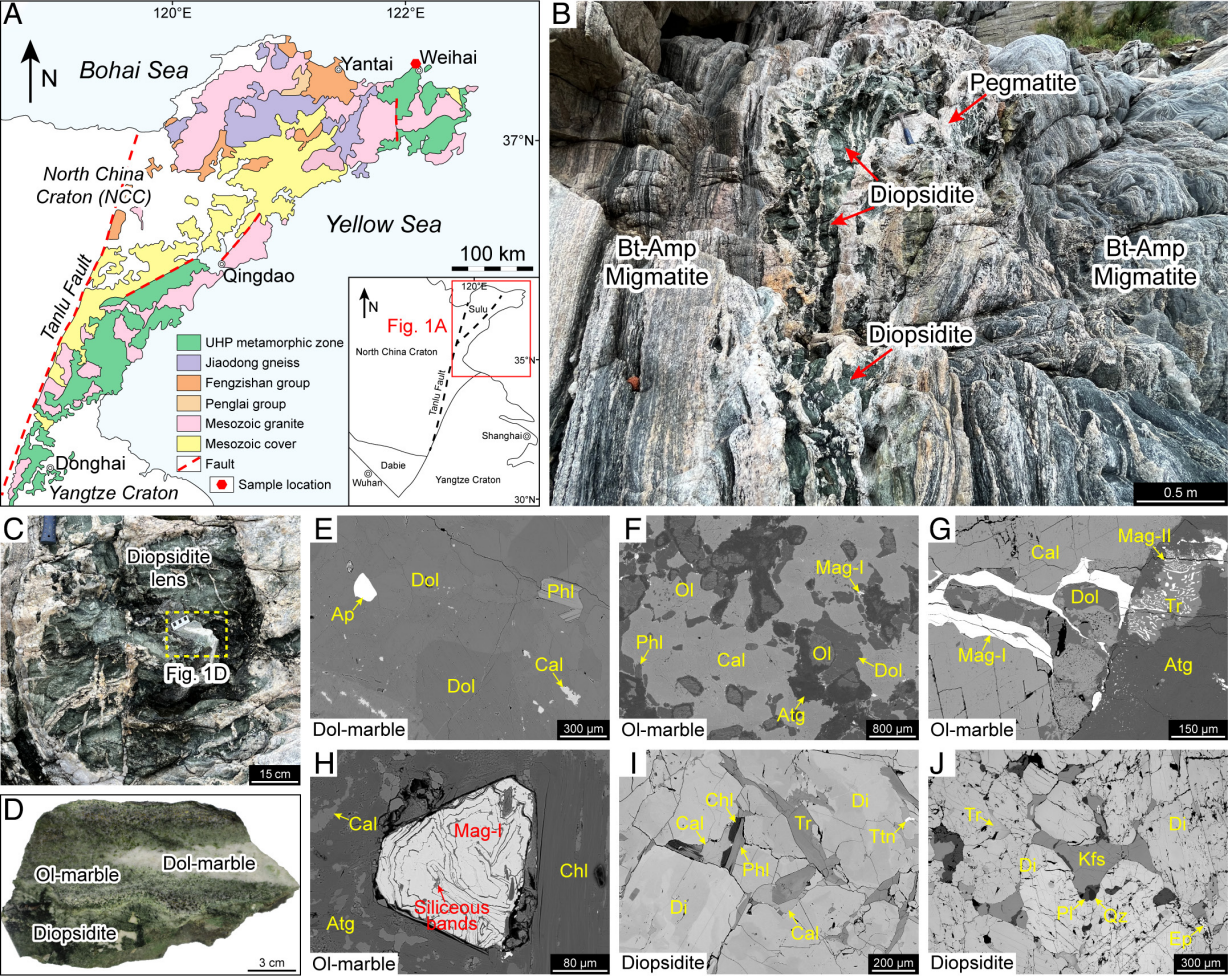
(*A*) Regional geological map of the Sulu UHP metamorphic belt in eastern China (modified from ref. 28). (*B*) Photograph showing diopsidite lenses in a deformed pegmatite dike. (*C*) A diopsidite lens that preserves a core of dolomitic marble and a mantle of olivine marble. (*D*) A hand specimen showing the core of dolomitic marble and the mantle of olivine marble in the diopsidite lens shown in (*C*). (*E*) Backscattered electron (BSE) image showing the mineral assemblage of dolomitic marble. (*F*–*H*) BSE images showing the mineral assemblages of olivine marble. (*G*) Film-like magnetite. (*H*) Granular magnetite that consists of rhythmically deposited siliceous bands and magnetite-rich bands. (*I* and *J*) BSE images showing the mineral assemblages of diopsidite. (*J*) Film-like assemblages of K-feldspar, plagioclase, and quartz with cuspate shapes.

The samples were collected from the Weihai region (Fig. 1*A*), a part of the Dabie-Sulu UHP belt that formed through the collision between the North China and Yangtse Cratons at approximately 220 to 240 Ma (21). The Weihai region predominantly consists of stromatic migmatites with abundant lenses of retrograde eclogite (granulite and amphibolite), ultramafic rock, and marble (22–32). The marble experienced decarbonation through interactions with fluids or partial melts, resulting in the formation of calc-silicate rocks (32). This research focuses on diopsidite lenses present in deformed pegmatite dikes in stromatic migmatites (Fig. 1*B*), sometimes with a core of dolomitic marble and a mantle of olivine marble (Fig. 1 *C* and *D*).

The dolomitic marble is almost pure and consists of dolomite (95 to 98 vol.%) with minor calcite, phlogopite, clinohumite, and apatite (<3 vol.%) (Fig. 1*E* and *SI Appendix*, Table S1). The olivine marble consists of calcite (30 to 60 vol.%) + dolomite (5 to 15 vol.%) + olivine (25 to 40 vol.%) + phlogopite (2 to 10 vol.%) + magnetite (Mag-I) (1 vol.%) + fluorite + clinohumite + sulfides (pyrite, chalcopyrite, sphalerite, and galena) ± diopside ± tremolite (Fig. 1 *F*–*H* and *SI Appendix*, Table S1), in which olivine is partly replaced by a late assemblage of antigorite ± clinohumite ± calcite ± dolomite ± talc ± magnetite (Mag-II) ± tremolite. Mag-I, with crystal sizes of 10 to 400 µm, occurs as film-like magnetite along the boundaries of calcite grains (Fig. 1*G*) and granular magnetite that consists of rhythmically deposited siliceous bands and magnetite-rich bands (Fig. 1*H*). The diopsidite consists of >90 vol.% diopside with subordinate tremolite, calcite, titanite, epidote, phengite, biotite/phlogopite, Mg-chlorite, apatite, plagioclase, K-feldspar, and quartz (Fig. 1 *I* and *J* and *SI Appendix*, Table S1). K-feldspar, plagioclase, and quartz occur as films with cuspate shapes that are interstitial to the framework of diopside grains (Fig. 1*J*), representing melt pseudomorphs (33).

**Fig. 2.**
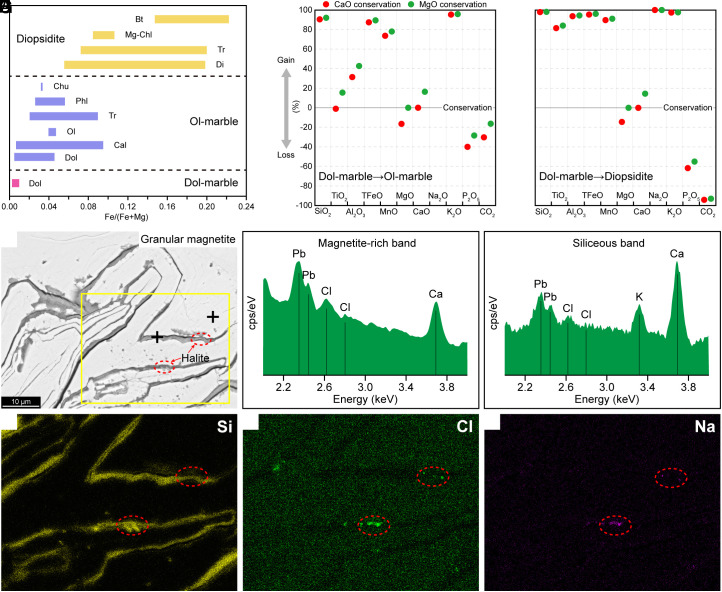
(*A*) Fe/(Fe + Mg) ratios of minerals from dolomitic marble, olivine marble, and diopsidite. (*B*) Mass balance calculations on the basis of CaO or MgO conservation showing mass gain and loss of components in olivine marble. (*C*) Mass balance calculations on the basis of CaO or MgO conservation showing mass gain and loss of components in diopsidite. (*D*) BSE image showing granular magnetite. (*E* and *F*) EDS analyses showing trace chlorine in magnetite-rich and siliceous bands. Analytical points are marked with plus symbols in image (*D*). (*G*–*I*) X-ray maps of Si, Cl, and Na showing halite grains in siliceous bands. The mapping area is marked with a yellow rectangle in image (*D*).

The transformations from dolomitic marble to olivine marble and diopsidite reflect mass gain of Si, Al, Fe, Na, K, F, Cl, and S and mass loss of C. The minerals in olivine marble and diopsidite have higher FeO contents and Fe/(Fe + Mg) ratios than the dolomite from the dolomitic marble (Fig. 2*A* and *SI Appendix*, Table S1), indicating a large mass gain of Fe. Mass balance calculations on the basis of Ca or Mg conservation reveal 30 to 95% mass gain of Si, Al, Fe, and K and 20 to 30% mass loss of CO_2_ for the formation of the olivine marble (Fig. 2*B* and *SI Appendix*, Tables S2 and S3) and >80% mass gain of Si, Al, Fe, Na, and K and nearly complete loss of CO_2_ for the formation of the diopsidite (Fig. 2*C* and *SI Appendix*, Tables S2 and S3). In addition, the influx of F, Cl, and S is indicated by the presence of fluorite, phlogopite, clinohumite, Cl-bearing tremolite, and sulfides in the olivine marble. The phlogopite and clinohumite have F contents of 0.19 to 1.07 and 2.02 to 2.37 wt.%, respectively, and the tremolite contains Cl contents of up to 0.20 wt.% (*SI Appendix*, Table S1). Moreover, granular magnetite contains trace amounts of Cl in magnetite-rich bands and siliceous bands, as shown in the EDS analyses (Fig. 2 *D*–*F*), and halite grains are identified in siliceous bands (Fig. 2 *G*–*I*).

**Fig. 3.**
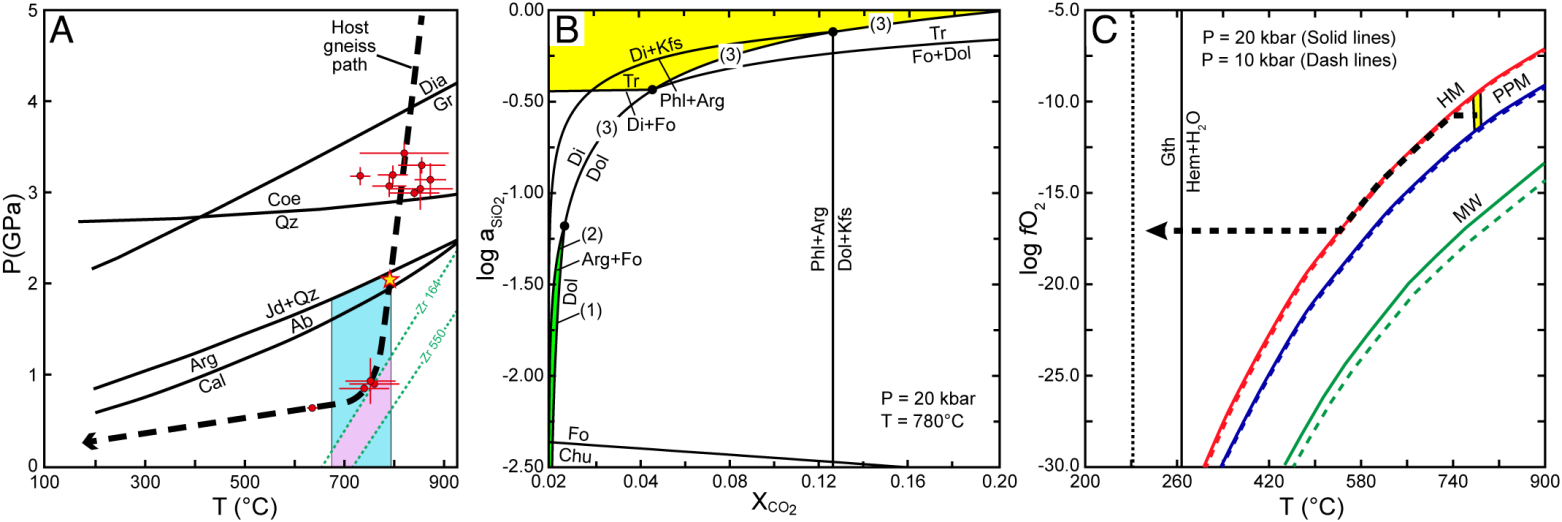
(*A*) P–T conditions for the formation of olivine marble and diopsidite. The P–T estimates of the Weihai UHP rocks (red circles with errors) are from refs. 23 and 24. The blue field represents the P–T conditions constrained by results from the calcite-dolomite solvus geothermometer and the stability fields of albite, calcite, and aragonite. The pink field represents the temperature range constrained by the results from the Zr-in-titanite thermobarometer and the calcite-dolomite solvus geothermometer. The green dotted lines represent the isopleths of the lowest and highest Zr contents in titanite (ppm). (*B*) Calculated decarbonation reactions for the formation of observed mineral assemblages in olivine marble and diopsidite in a_SiO2_–X_CO2_ space at 780 °C and 2.0 GPa. Olivine marble and diopsidite are stable in the green and yellow fields, respectively. (*C*) The oxygen fugacity during the decarbonation process (yellow area). A decrease in temperature during the exhumation process can result in the transformation of magnetite into hematite and subsequently into goethite within fluid-bearing inclusions.

The P–T conditions for the olivine marble and diopsidite formation are constrained with the calcite–dolomite solvus geothermometer (34), the Zr-in-titanite thermobarometer (35), and inclusions in diopsidite zircon. The calcite grains in olivine marble contain 11 to 22 vol.% exsolved dolomite lamellae (*SI Appendix*, Fig. S1), yielding temperatures of 670 to 800 °C on the basis of the integrated compositions determined from the mineral compositions and mineral modal proportions (*SI Appendix*, Table S4). Aragonite, calcite, and albite were identified in the inclusions in zircons from diopsidite (Table 1), suggesting that the decarbonation reactions occurred from the aragonite + albite to calcite stability fields (Fig. 3*A*). The Zr contents of titanite from the diopsidite range from 164 to 550 ppm (*SI Appendix*, Fig. S2 and Table S5). Below a pressure of 1.0 GPa, the Zr-in-titanite thermobarometer yields temperature estimates that are consistent with those obtained from the calcite–dolomite solvus geothermometer (Fig. 3*A*).

**Table 1.** Representative assemblages of inclusions in zircons from olivine marble and diopsidite

Inclusion types	Whe-bearing inclusions (H_2_O/CO_2_)	DCM-bearing inclusions (H_2_O/CO_2_/CH_4_/H_2_)	Volatile-bearing inclusions (H_2_O/CO_2_/CO/H_2_)	Mineral inclusions
Olivine marble	Whe+DCM+Cal+CO_2_+H_2_O (4-7-1)	DCM+Phl+Mgs+Cv (1-13-1), DCM (2-9-1), DCM+Phl+H_2_+CH_4_ (3-19-1), DCM+Phl (4-9-1)	CO_2_ (1-6-1), CO_2_+Mgs (1-11-1), Dol+CO_2_ (3-11-1), Ol+CO_2_ (3-12-1), Gth+H_2_O (3-17-2), Phl+H_2_ (3-19-2), Mag+Brk+H_2_ (4-10-1)	Ol+Di+Rt (1-2-1), Ol (1-3-1), Dol (1-5-1), Gth (1-6-1), Bdy(1-7-1), Chu (1-9-1), Dol+Cal (1-10-1), Dol+Phl (2-2-1), Brk (2-2-2), Di (2-13-1), Dol+Mag+Hem (2-14-1), Cal (2-16-1), Mgs (2-17-1), Phl+Ol (3-15-3), Phl+Mag+Hem (4-50-1)
Diopsidite	Whe+CO_2_ (1-70-4), Whe+DCM+Cal+Ms+Ccp+CO_2_+H_2_O (1-144-1), Whe+DCM+CO_2_ (2-49-3), Whe+Ms+H_2_O (2-143-2)	DCM+Ccp+Ms (1-1-1), DCM (1-5-1), DCM+Mag+Hem+Kfs (1-5-10), DCM+Gth (1-42-1), DCM+Mag+Hem+Gth+Phl+Cal (1-45-1), DCM+Cal (1-48-11), DCM+CH_4_+Cal+Crs (1-48-6), DCM+Mag+Hem (1-50-8), DCM+Mag+Gth (1-50-10), DCM+Ap (1-52-7), DCM+H_2_+Cal (1-79-1), DCM+Vtr (1-79-11), DCM+CO_2_+Kdy (1-79-13), DCM+Hem+Gth (1-93-9), DCM+Ms+Kfs+Hem (1-93-10), DCM+H_2_+Qz (1-98-1), DCM+Di+Gth (1-115-5), DCM+Cal+Crs+Ap (1-114-2), DCM+CO_2_+H_2_O (1-147-2), DCM+Mnz+Gth (2-40-1), DCM+Gth+Ab+Qz (2-42-1), DCM+Ms (2-49-2), DCM+Cal+Qz+CO_2_+H_2_O (2-143-1)	Cal+Crs+CO_2_+H_2_O (1-48-8), Cal+CO_2_ (1-48-14), CO_2_+H_2_O (1-69-9), Qz+CO_2_ (1-70-3), Ms+CO_2_ (1-77-2), CO_2_ (1-77-3), Ms+CO_2_+H_2_O (1-77-6), H_2_+H_2_O (1-79-2), H_2_+Cal (1-79-5), H_2_+Qz (1-79-7), H_2_ (1-79-19), Qz+CO_2_+CO (1-79-10), H_2_+Qz+H_2_O (1-98-3)	Gth (1-1-2), Cal (1-5-3), Ms (1-5-5), Phl (1-5-11), Ap (1-5-6), Cal+Ms (1-42-11), Qz (1-44-2), Mag+Hem+Qz (1-45-2), Mag+Hem (1-45-5), Tr (1-45-7), Hem (1-48-17), Cal+Qz+Ms (1-48-2), Cal+Crs (1-48-10), Qz+Ap (1-48-4), Gp (1-48-9), Ant (1-48-18), Kct (1-50-13), Cal+Qz+Brt (1-52-3), Kfs+Ms (1-52-1), Kfs (1-52-2), Mag+Hem+Cal (1-69-3), Ttn (1-69-8), Ab (1-72-3), Di (1-77-4), Arg+Qz+Cun (1-79-4), Vtr+Qz (1-79-16), Hem+Kfs (1-95-5), Hem+Mag+Kfs (1-95-10), Vtr (1-98-6), Cal+Ms+Qz+Gp (1-111-2), Cun (1-116-6), Cal+Glass (1-114-3), Cal+Ap (1-144-9), Brt (1-144-7), Anh+Ap (1-144-13), Mag+Hem+Tr+Cal+Ms (2-49-1), Gth+Di (3-18-5), Gth+Qz (3-18-10), Di+Qz (3-18-4), Gth+Ant+Phl (3-20-3), Brk (3-20-5)

The number in parentheses is inclusion No.

The transformations of the dolomitic marble to olivine marble and diopsidite suggest the following reactions for the formation of olivine, phlogopite, diopside, and calcite/aragonite, accompanied by CO_2_ release:

[1] $$2\mathrm{Dol}+{\mathrm{SiO}}_{2}(\mathrm{aq})=\mathrm{Fo}+2\mathrm{Cal}/\mathrm{Arag}+2{\mathrm{CO}}_{2}$$

[2] $$\mathrm{Fo}+2\mathrm{Cal}/\mathrm{Arag}+3{\mathrm{SiO}}_{2}(\mathrm{aq})=2\mathrm{Di}+2{\mathrm{CO}}_{2}$$

[3] $$\mathrm{Dol}+2{\mathrm{SiO}}_{2}(\mathrm{aq})=\mathrm{Di}+2{\mathrm{CO}}_{2}$$

[4] $$\begin{array}{c} 6\mathrm{Dol}+K_{2}O(\mathrm{aq})+{\mathrm{Al}}_{2}O_{3}(\mathrm{aq})+6{\mathrm{SiO}}_{2}(\mathrm{aq})+2H_{2}O\\ \;=2\mathrm{Phl}+6\mathrm{Cal}/\mathrm{Arag}+6{\mathrm{CO}}_{2} \end{array}$$

These reactions are controlled by SiO_2_ activity (a_SiO2_) and X_CO2_ in reactive fluids under given P–T conditions. Reference conditions of 780 °C and 2.0 GPa were selected to calculate the stability fields for the observed mineral assemblages, which included aragonite, olivine, phlogopite, diopside, tremolite, K-feldspar, and quartz in a_SiO2_–X_CO2_ space in a simplified Fe-free system, using the thermodynamic dataset (36) and Perplex_X 6.9.1 (37). Fig. 3*B* shows that the olivine + aragonite assemblage is equilibrated with aqueous SiO_2_-poor fluids with log a_SiO2_ <−1.2 (a_SiO2_ = 0.06) and X_CO2_ <0.01, whereas the diopside + tremolite and diopside + phlogopite assemblages in the diopsidite are equilibrated with SiO_2_-rich fluids or hydrous melt with log a_SiO2_ >−0.45 (a_SiO2_ >0.35).

The redox state in the decarbonation process may be constrained by the presence of magnetite and pyrite in olivine marble as the following equilibria:

[5] $$4{\mathrm{Fe}}_{3}O_{4}(\mathrm{magnetite})+O_{2}=6{\mathrm{Fe}}_{2}O_{3}(\mathrm{hematite})$$

(HM)

[6] $$\begin{array}{c} 3{\mathrm{FeS}}_{2}(\mathrm{pyrite})+{\mathrm{Fe}}_{3}O_{4}(\mathrm{magnetite})+4H_{2}\\ \;=6\mathrm{FeS}(\mathrm{pyrrhotite})+4H_{2}O \end{array}$$

(PPM)

The oxygen fugacity (log ƒO_2_) was between the HM and PPM buffers, and the calculation using Perplex_X 6.9.1 (37) yields a range between −9 and −12 at 2.0 GPa and 780 °C (Fig. 3*C*).

### Inclusions in Zircon.

**Fig. 4.**
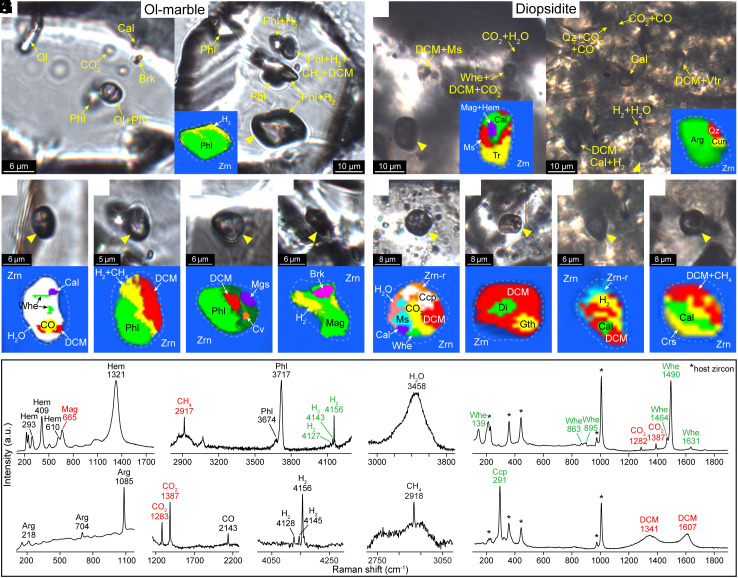
Photomicrographs (in plane-polarized light) and Raman maps showing the inclusions in zircons from olivine marble (*A*–*E*) and diopsidite (*F*–*J*). Whewellite, DCM, CO, CH_4_, H_2_, CO_2_, H_2_O, and the minerals from decarbonation reactions are present in the inclusions in zircons from olivine marble and diopsidite. (*K*) Representative Raman spectra of phases in the inclusions. Zrn-r–zircon with different crystalline orientation from the host zircon.

Zircon, as a refractory and mechanically robust mineral, can resist later chemical and physical changes and thus is a good container for inclusion preservation. The inclusions in zircons from the olivine marble and diopsidite were identified using Raman spectroscopy. The zircons from the olivine marble, with sizes ranging from 40 to 100 μm, contain sporadic isolated inclusions (Fig. 4*A*), whereas the zircons from the diopsidite, with sizes ranging from 100 to 200 μm, feature a core–rim structure characterized by a patchy and mosaic core with abundant inclusions and an overgrowth oscillatory rim without inclusions in CL images (Fig. 4*F* and *SI Appendix*, Fig. S3*A*). SIMS U–Pb dating of zircons from the diopsidite shows that 22 analyses of the cores yield a Concordia age of 218.5 ± 1.9 Ma, whereas 24 analyses of the rims yield a Concordia age of 218.3 ± 1.9 Ma (*SI Appendix*, Fig. S3*B* and Table S6).

On the basis of the presence of whewellite, disordered carbonaceous material (DCM), and volatiles, the inclusions are divided into whewellite-bearing inclusions, DCM-bearing inclusions (without whewellite), volatile-bearing inclusions (without whewellite or DCM), and mineral inclusions (without whewellite, DCM, or volatiles) (Table 1). The whewellite-bearing inclusions in zircon from the olivine marble are fluid inclusion with whewellite, DCM, calcite, CO_2_, and H_2_O (Fig. 4*B* and Table 1). The DCM-bearing inclusions include phlogopite, magnesite, covellite, H_2_, and CH_4_ (Fig. 4 *A*, *C*, and *D* and Table 1). The volatile-bearing inclusions contain H_2_O, CO_2_, H_2_, and minerals such as olivine, phlogopite, calcite, magnesite, dolomite, magnetite, goethite, and brookite (Fig. 4 *A* and *E* and Table 1). The mineral inclusions include olivine, phlogopite, clinohumite, diopside, dolomite, calcite, magnesite, magnetite, hematite, goethite, rutile, brookite, and baddeleyite (Fig. 4*A* and Table 1). The whewellite-bearing inclusions in zircon from the diopsidite are fluid inclusions with H_2_O, CO_2_, DCM, calcite, muscovite, and chalcopyrite (Fig. 4 *F* and *G* and Table 1). The DCM-bearing inclusions include magnetite, hematite, goethite, chalcopyrite, calcite, vaterite, albite, kumdykolite, K-feldspar, kokchetavite, quartz, cristobalite, diopside, phlogopite, apatite, calciouranoite, and volatiles H_2_O, CO_2_, H_2_, and CH_4_ (Fig. 4 *F* and *H–J* and Table 1). The volatile-bearing inclusions consist of H_2_O, CO_2_, CO, H_2_, and minerals such as magnetite, hematite, goethite, calcite, quartz, cristobalite, muscovite, apatite, and anhydrite (Fig. 4*F* and Table 1). The mineral inclusions are either multiphase or single-phase inclusions, including magnetite, hematite, goethite, calcite, vaterite, aragonite, apatite, diopside, tremolite, phlogopite, muscovite, K-feldspar, kokchetavite, albite, quartz, silicic glass, gypsum, anhydrite, barite, anatase, brookite, and calciouranoite (Fig. 4*F* and Table 1).

The Raman spectra of some materials in the inclusions are shown in Fig. 4*K*. Whewellite displays two strong bands at 1,490 and 1,464 cm^−1^, medium to weak bands at 1630, 896, and 140 cm^−1^, and a broad band of H_2_O molecule from 3,000 to 3,600 cm^−1^ (Fig. 4*K*; ref. 38). DCM is a graphite-like carbonaceous material with I_D_/I_G_ ratios of 0.39 to 1.10. The D band is centered at ~1,328 to 1,349 cm^−1^ with a full width at half maximum (FWHM) of 71 to 110 cm^−1^, and the G band is centered at 1,578 to 1,599 cm^−1^ with a FWHM of 52 to 101 cm^−1^ (*SI Appendix*, Fig. S4 and Table S7) on the basis of the band-fitting results with additional bands at ~1,620 cm^−1^ (D2), ~1,510 cm^−1^ (D3), ~1,250 cm^−1^ (D4), ~1,150 cm^−1^ (D5), and ~1,440 cm^−1^ (D6) (39, 40).

**Fig. 5.**
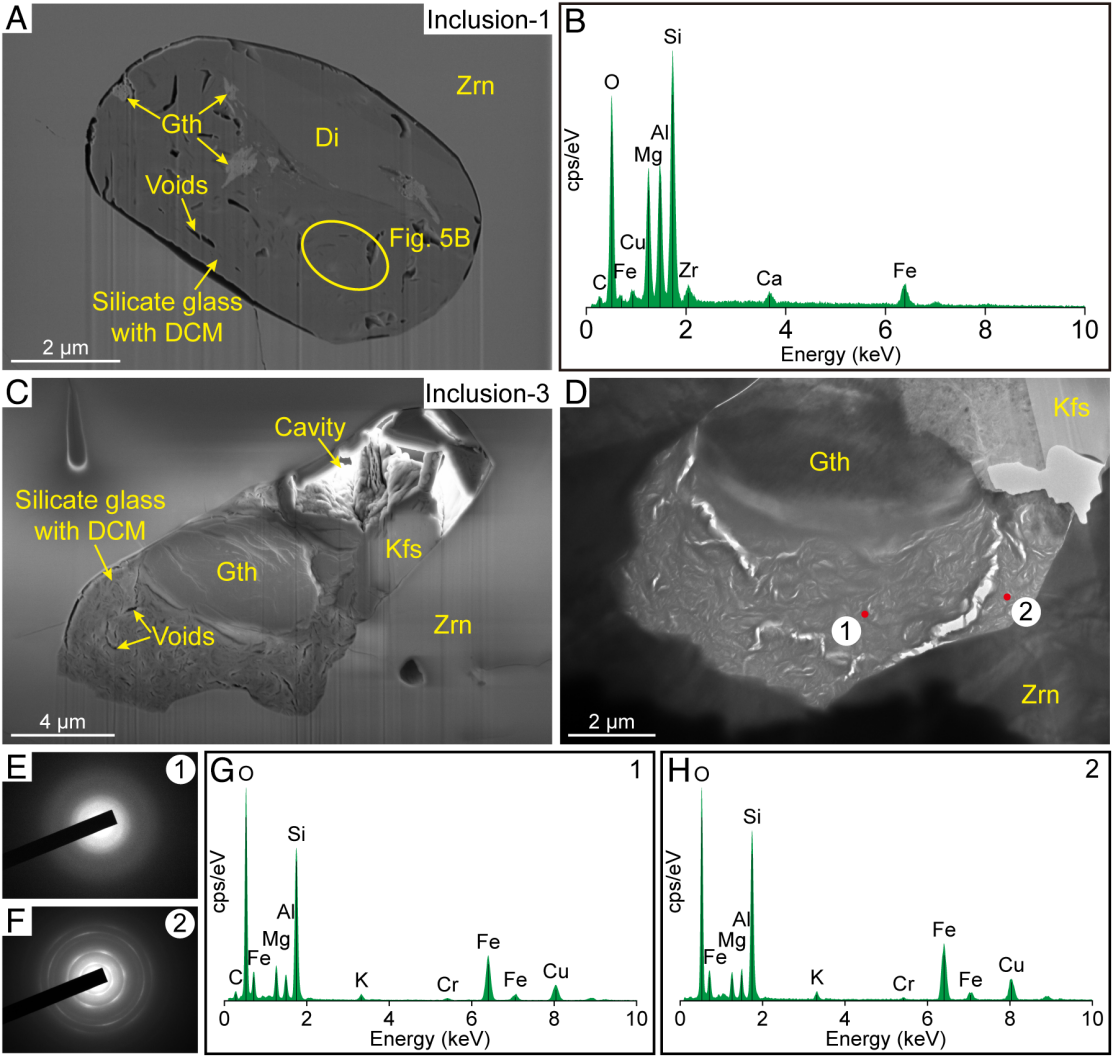
(*A*) Secondary electron (SE) image of a FIB-milled vertical cross-section showing a DCM-bearing inclusion (inclusion-1) in zircon from diopsidite. (*B*) X-ray compositional spectrum of silicate glass in inclusion-1. (*C*) SE image of a FIB-milled vertical cross-section showing a DCM-bearing inclusion (inclusion-3) in zircon from diopsidite. (*D*) Bright-field image showing the silicate glass in inclusion-3. (*E*) Selected-area electron diffraction (SAED) image of domain 1 in (*D*), indicating that the material is completely amorphous. (*F*) SAED image of domain 2 in (*D*), revealing the presence of nano-sized crystals. (*G* and *H*) X-ray compositional spectra of domains 1 and 2 marked in (*D*).

Several DCM-bearing inclusions were observed using focused ion beam–scanning electron microscopy (FIB-SEM) and transmission electron microscopy (TEM) (Fig. 5). The DCM-bearing inclusions consist of large cavities and silicate glass with abundant micron-sized elongated voids and minerals (Fig. 5 *A*, *C*, and *D*), sometimes with radiating fractures and offshoots. The silicate glass, with or without nano-sized undetermined minerals (Fig. 5 *E* and *F*), consists of Mg, Al, Si, and O with variable Fe, Ca, Zr, and K, and DCM occurs in the glass (Fig. 5 *B*, *G* and *H*).

## Discussion

### Evidence for Abiotic Synthesis.

The presence of whewellite and DCM suggests that the primary inclusions contained organic dicarboxylic compounds that reacted with dissolved CaCO_3_ to produce whewellite during the exhumation process of the rocks (31). The DCM shows several Raman subbands, including the D4 band (~1,150 cm^−1^), the D5 band (~1,260 cm^−1^), and the D6 band (~1,380 to 1,430 cm^−1^) (*SI Appendix*, Fig. S4*A*), which originate from the CH vibrations of aliphatic components (39, 40). On the basis of calculations from the integrated intensity ratio I_D5_/(I_G_ + I_D2_) (39), the DCM has H:C ratios of 0.28 to 0.69 (*SI Appendix*, Table S7). Therefore, the DCM is inferred to have originated from the incomplete carbonization of some kind of organic matter (e.g., the inferred dicarboxylic polymers) during the postentrapment re-equilibration (31).

The whewellite-bearing and DCM-bearing inclusions are associated with or coexist with volatiles (H_2_O, CO_2_, CO, CH_4_, and H_2_), minerals from decarbonation reactions (olivine, phlogopite, diopside, tremolite, and calcite; Fig. 3*B*), and quartzo-feldsparthic minerals from infiltrating partial melts (albite, kumdykolite, K-feldspar, kokchetavite, quartz, and silicate glass; *SI Appendix,* Fig. S5), suggesting that the inclusions were entrapped during the interaction of the dolomitic marble with immiscible fluid and melt from the partial melting of country gneisses. The inclusions occur in the core parts of zircons from the diopsidite, indicating that they were trapped in the early stage of zircon growth. Several studies on eclogites and their country migmatites in the Weihai region have shown that coesite-bearing zircons yield consistent UHP ages ranging from 238 to 220 Ma, whereas leucosomes and pegmatites in migmatites crystallized at 220 to 210 Ma after the UHP metamorphism (25, 26, 28, 29, 31, 41–43). SIMS U–Pb analyses of the zircons in this study revealed that the inclusions were trapped at 218.5 ± 1.9 Ma (*SI Appendix*, Fig. S3*B*), indicating that the decarbonation of the dolomitic marble occurred during the exhumation process of the UHP rocks. The temperature and pressure of decarbonation are consistent with the retrograde granulite-facies conditions recorded in the Weihai eclogites, with pressures ranging from 0.7 to 1.2 GPa and temperatures ranging from 700 to 850 °C (22–24, 32). Under such high-grade metamorphic conditions, biotic organic compounds cannot be preserved due to their carbonization and graphitization (18, 19). Therefore, the organic matter indicated by the presence of whewellite and DCM reveals the occurrence of abiotic synthesis during the decarbonation process of the dolomitic marble.

### Mechanism of Abiotic Synthesis.

In observed rock systems such as serpentinized ultramafic rocks and altered alkaline igneous rocks, Fe is the key element for the generation of H_2_. Fe is commonly considered to be from the Fe^2+^ in minerals in rocks, which reacts with water to produce H_2_ and magnetite. The presence of organics in these altered rocks is attributed to FTT reactions among magnetite, CO_2_, and H_2_. The system with magnetite, CO_2_, and H_2_ is also formed by other reactions, e.g.,

[7] $$3{\mathrm{FeCO}}_{3}+H_{2}O={\mathrm{Fe}}_{3}O_{4}+H_{2}+3{\mathrm{CO}}_{2}$$

This reaction is also accompanied by the generation of organic compounds (2, 44, 45). Therefore, the formation of magnetite, CO_2_, and H_2_ through fluid–rock interactions is crucial for the abiotic synthesis of organic compounds in natural rocks.

The dolomitic marble, as the precursor rock, has a very low Fe content (0.25 wt.% TFe_2_O_3_; *SI Appendix,* Table S2), and the FeCO_3_ endmember in the dolomite is less than 0.90 mol.% (*SI Appendix,* Table S1). The Fe enrichment in olivine marble and diopsidite (2.06 wt.% and 4.28 wt.% TFe_2_O_3_, respectively; *SI Appendix,* Table S2) occurred during the decarbonation of dolomitic marble. Mass balance calculations based on Ca or Mg conservation yield CO_2_ mass losses of 16 to 30% for the olivine marble and 93 to 94% for the diopsidite (Fig. 2 *B* and *C* and *SI Appendix,* Table S3), whereas calculations based on Fe conservation give CO_2_ mass losses of 91.1% for the olivine marble and of 99.7% for the diopsidite (*SI Appendix,* Table S3). The results from Ca or Mg conservation are consistent with the petrological observations that olivine marble contains 35 to 75 vol.% calcite and dolomite (Fig. 1*F*) and diopsidite contains 2 to 3 vol.% calcite (Fig. 1*I*), but the results based on Fe conservation do not. Therefore, the Fe enrichment in olivine marble and diopsidite requires the external introduction of Fe through infiltrating fluids, with minimum fluid/rock ratios of 2 to 9 (*SI Appendix*, Supporting Text). In addition, magnetite displays evidence of precipitation from the fluid phase. The rhythmically deposited bands in the granular magnetite (Fig. 1*H*) were originated from periodical precipitation from the fluid phase, and the presence of trace chlorine and halite in the siliceous bands indicates that the fluids were Fe chloride–rich silicic brines (Fig. 2 *D*–*I*). Such silicic concentrated brines have extraordinary wetting ability (46), which can occur as fluid films along the boundaries of minerals. The film-like magnetite (Fig. 1*G*) should be the result of mimetic precipitation along the fluid films.

Magnetite and pyrite occur as precipitates in the matrix of the olivine marble, indicating that the oxygen fugacity of the infiltrating fluids was between the HM and PPM buffers (Fig. 3*C*). Experiments on Fe solubility and speciation in chloride-bearing supercritical fluids under such redox conditions have shown that ferrous chloride species, such as FeCl_2_ (15, 47), octahedral FeCl_x_(H_2_O)_6−x_^2−x^ (x = 0 to 3), and tetrahedral FeCl_4_^2−^or FeCl_3_(H_2_O)^−^ (14), are predominant.

Ferrous chloride fluid can react with CaCO_3_ via the following equilibrium (15):

[8] $$\begin{array}{c} 3{\mathrm{FeCl}}_{2}(\mathrm{aq})+3{\mathrm{CaCO}}_{3}+H_{2}O\\ \;={\mathrm{Fe}}_{3}O_{4}(\mathrm{magnetite})+3{\mathrm{CaCl}}_{2}(\mathrm{aq})+3{\mathrm{CO}}_{2}+H_{2} \end{array}$$

This reaction is responsible for the formation of magnetite deposits in skarns (15). The inclusions in zircons include magnetite, calcite/aragonite, H_2_O, CO_2_, and H_2_, confirming that reaction [8] was the decarbonation and dehydrogenation reaction that occurred in the olivine marble and diopsidite. Because the Fe^3+^ in olivine marble and diopsidite was predominantly produced by reaction [8], the amount of H_2_ produced during metasomatism can be estimated on the basis of the Fe_2_O_3_ contents in the two rocks (1.15 wt.% in olivine marble and 2.26 wt.% in diopsidite; *SI Appendix*, Supporting Text and Table S2). Metasomatism could have produced 72 mmol H_2_ per kg of olivine marble and 142 mmol H_2_ per kg of diopsidite, which is comparable to the amount of H_2_ released during the serpentinization of ultramafic rocks, which ranges from 13 to 315 mmol/kg H_2_ (48, 49).

In addition, pyrite precipitation from ferrous chloride fluid is another H_2_-producing reaction as the following equilibrium (50):

[9] $$\begin{array}{c} {\mathrm{FeCl}}_{2}(\mathrm{aq})+2H_{2}S(\mathrm{aq})+H_{2}O\\ \;={\mathrm{FeS}}_{2}(\mathrm{pyrite})+H_{2}+2H^{+}+2{\mathrm{Cl}}^{-} \end{array}$$

Because the modal proportion of pyrite is very low in the rocks, this reaction is unlikely to have contributed significantly to H_2_ production.

The H_2_-induced reduction of CO_2_ released from decarbonation reactions can produce CO and CH_4_ present in the inclusions as the following reactions (2):

[10] $${\mathrm{CO}}_{2}+H_{2}=\mathrm{CO}+H_{2}O$$

[11] $${\mathrm{CO}}_{2}+4H_{2}={\mathrm{CH}}_{4}+2H_{2}O$$

Moreover, the presence of whewellite in the inclusions indicates that complex carboxylic groups were involved in the decarbonation process. However, the dicarboxylic compound in our sample could not possibly have been oxalic acid or oxalate since the metamorphic temperature was far beyond the stability field of oxalic acid or oxalate. Some dicarboxylic polymers have high P–T stability fields (51) and thus have been inferred to be plausible candidates for the organic compounds in the primary fluid inclusions (31).

To date, studies on H_2_-producing processes in natural rocks have focused mainly on low-temperature, low-pressure (<400 °C, <0.5 GPa) serpentinized ultramafic rocks and altered alkaline igneous rocks (1–3, 10). However, H_2_ can also be produced during high-temperature, high-pressure (450 to 600 °C, 1.0 to 2.0 GPa) antigorite serpentinization (49, 52, 53). Recent studies have found H_2_ and abiotic hydrocarbons present in fluid inclusions within the UHP carbonate-bearing eclogites from the southwestern Tianshan subduction zone (45, 54), where H_2_ was generated via reaction [7]. Our results reveal that the H_2_-producing reaction and FTT process can occur under higher-temperature conditions (670 to 800 °C), and H_2_ in this study was mainly formed via reaction [8] rather than reaction [7] due to the negligible Fe content in dolomite (*SI Appendix,* Table S1). In addition, the identification of organic species (hydrocarbons and carboxylic functional groups) in the fluid inclusions in diamonds, garnets, and zircons suggests that abiotic geosynthesis of organic compounds occurred under the mantle conditions at depths >100 km (31, 55, 56), meaning that H_2_ should also be generated during those metamorphic processes. Abiotic geosynthesis of organic compounds should prevail under elevated P–T conditions because high-molecular-weight hydrocarbons and aliphatic acids are thermodynamically stable under such conditions (45, 57–61). However, during contact metamorphism, skarns, which generally form at pressures <0.3 GPa, commonly contain CH_4_ (62) but lack high-molecular-weight hydrocarbons and aliphatic acids in fluid inclusions. Some studies have suggested that CH_4_ in skarns is abiotically produced (63–65). The lack of high-molecular-weight hydrocarbons and aliphatic acids in skarns should be related to low-pressure conditions.

### Concluding Remarks.

The decarbonation of marbles induced by infiltrating fluids and melts is common in metamorphic circumstances (32, 66–69). This study shows that aqueous Fe^2+^–Cl species in infiltrating fluids reacted with CaCO_3_ minerals to produce magnetite, CO_2_, and H_2_ (15), and the presence of whewellite and DCM in the inclusions of zircons verifies that magnetite-catalyzed FTT synthesis synchronously occurred during the reaction process. Since brines and marbles are common in a variety of metamorphic circumstances (15, 70–72), organic synthesis during the decarbonation process should occur widely. Marble decarbonation in this study occurred under granulite-facies conditions, indicating that subduction zones and orogenic roots with high P–T metamorphic conditions are suitable sites for abiotic synthesis.

## Materials and Methods

### Sample Preparation and Zircon CL Imaging.

Zircons were separated from the samples using magnetic techniques. The separates were handpicked, embedded in epoxy, and polished to approximately half of their thickness. Cathodoluminescence (CL) images of zircon grains were obtained at the Institute of Geology and Geophysics, Chinese Academy of Sciences (IGGCAS), using a Nova NanoSEM 450 field emission scanning electron microscope (SEM) equipped with a Gatan MonoCL4 cathodoluminescence spectrometer.

### Major Element Analyses of Minerals.

A JEOL JXA-8100 electron probe at the IGGCAS was used to analyze the mineral compositions. The analyses were performed in wavelength-dispersion mode with an acceleration voltage of 15 kV, a beam current of 10 nA, and a beam diameter of 1 μm. Natural and synthetic oxides were used as standards, and the precision was better than 1.5% (1σ). X-ray composition map and energy-dispersive X-ray spectroscopy (EDS) data were acquired by using a Zeiss Gemini 450 field-emission SEM instrument equipped with an X-ray energy dispersive spectrometer at the IGGCAS with a 15 kV acceleration voltage and 2 nA beam current.

### Whole-Rock Contents of Major Elements and Ferrous Iron.

Bulk analyses of major elements were conducted at the IGGCAS. Rock samples were first ground into a fine powder using an agate mortar. Approximately 0.5 g of sample powder was mixed with 5 g of Li_2_B_4_O_7_ powder, heated and fused into a glass disk, and then analyzed using a PANalytical AXIOS XRF spectrometer. Analyses of rock reference materials GBW07114 and GBW07120 indicate that the analytical uncertainties range from 1 to 3% for elements present at >1 wt.% and approximately 10% for elements <1 wt.%. For ferrous iron, approximately 0.2 g of sample powder was weighed by determining the difference in the PTFE vessel. The powder was then digested with 10 mL of 50% H_2_SO_4_ and 5 ml of HF on a hot plate at 170 °C for 10 min. The obtained solution was complexed with 50 mL of saturated boric acid. Ferrous iron was titrated with a standard potassium permanganate solution (0.1119 mol/L). The detailed method was described by ref. 73. The accuracy of the ferrous iron contents was estimated to be better than ±10% on the basis of repeated analyses of the silicate standards (GSR-1 and GSR-2).

### Zr Analyses of Titanite.

The Zr contents of titanite were obtained using laser ablation inductively coupled plasma mass spectrometry (LA–ICP–MS) with an excimer 193 nm (ArF) resolution nanosecond laser ablation system (Australian Scientific Instruments) coupled with a quadrupole inductively coupled plasma mass spectrometer (8900 ICP–MS/MS, Agilent, USA) at the IGGCAS. After a warmup of the 8900 ICP–MS/MS instrument and connection with the laser ablation system, the instrument was first tuned for robust plasma conditions by optimizing the laser and ICP–MS/MS settings and monitoring the ^232^Th^16^O^+^/^232^Th^+^ ratios (always ≤0.5%) and ^238^U^+^/^232^Th^+^ ratios (always between 0.90 and 1.10) while ablating NIST SRM 610 in line scan mode. The analyses were performed using a spot diameter of 38 µm, a repetition rate of 5 Hz, and a laser energy density of 3.0 J·cm^−2^. Each analysis included 20 s of background acquisition and 70 s of data acquisition, with a total dwell cycle of 320 ms, including a 6 ms dwell time for ^90^Zr. The element contents were calibrated using an NIST 610 silicate glass wafer as the external calibrant, combined with internal standardization using ^29^Si. The raw intensities were processed using Iolite v4.0 software (74), which uses a data reduction scheme for trace element quantification. The accuracy for ^90^Zr was better than 2% (2σ).

### SIMS U–Pb Dating of Zircon.

Measurements of the U, Th, and Pb isotopes of zircons were conducted using a Cameca IMS-1280HR SIMS at the IGGCAS. Zircon grains with zones large enough for dating were selected for analysis. The primary O_2_^−^ ion beam spot was approximately 10 × 15 μm in size. Positive secondary ions were extracted with a 10 kV potential. Analyses of the standard zircon Plesovice were interspersed with those of unknown grains. Pb/U calibration was performed relative to the zircon standard Plesovice (^206^Pb/^238^U age = 337 Ma; ref. 75); U and Th concentrations were calibrated against the zircon standard 91500 (Th = 29 ppm, and U = 81 ppm; ref. 76). The measured compositions were corrected for common Pb using nonradiogenic ^204^Pb. The corrections were sufficiently small to be insensitive to the choice of common Pb isotopic composition. An average of present-day crustal composition (77) was used for common Pb, assuming that the common Pb is largely surface contamination introduced during sample preparation. Data reduction was carried out using the Isoplot/Ex v. 2.49 program (78). Uncertainties in individual analyses in the data tables are reported at 1σ level. To monitor the external uncertainties of the SIMS U–Pb zircon dating calibrated against the Plesovice standard, an in-house zircon standard, Qinghu, was alternately analyzed as an unknown zircon together with other unknown zircons. Twenty-four measurements on Qinghu zircon yield a Concordia age of 159.3 ± 0.5 Ma, which is identical within error with the recommended value of 159.5 ± 0.2 Ma (79). The detailed analytical procedures are described by (80).

### Raman Analysis.

Raman analysis of the inclusion was performed using a WITec confocal Raman microscope alpha 300R at the IGGCAS. Raman spectra were acquired using a diode-pumped solid-state laser (532 nm, cobalt laser) and a thermoelectrically cooled CCD detector. Analyses were performed at 1 to 12 mW laser power with a 100× objective (numerical aperture = 0.90) at 300 and 1,800 grooves/mm gratings. The 300 grooves/mm grating was mainly used to map the inclusions because it allowed us to obtain a spectrum from 100 to 4,000 cm^−1^ in a spectral window. An 1,800 grooves/mm grating was used to obtain spectra with a higher spectral resolution of approximately 0.8 cm^−1^. Raman mapping was generated with a step size of 0.3 μm, an acquisition time of 2 to 5 s, and one accumulation. The Raman system was calibrated using the emissions of an Ar–Hg lamp and the Rayleigh line. Raman bands of the analyzed minerals were identified on the basis of the RRUFF database (81) and refs. 82 and 83.

### FIB-SEM Analysis.

FIB foils were prepared using a Zeiss Auriga Compact dual beam system with a Ga-ion beam at the IGGCAS. The conditions for milling were 5 to 30 kV with a beam current of 12 to 500 pA. SE images were acquired at an accelerating voltage of 1.5 to 10 kV. No coating was applied to the foil. The foils were milled to a thickness of approximately 100 nm for TEM observation.

### TEM Analysis.

The FIB-prepared foils of inclusions were observed using a JEOL JEM-2100 transmission electron microscope with an Oxford X-MAX energy dispersive EDS at 200 kV at the IGGCAS. The illumination area for electron diffractions was approximately 130 nm, and a SAED image was taken at a camera length of 30 cm. EDS analysis was performed at 200 kV with a beam current of 106 μA and a beam size of 5 to 10 nm using Oxford Aztec software.

## Supplementary Material

Appendix 01 (PDF)

## Data Availability

All study data are included in the article and/or *SI Appendix*.
