# Supplementary material for: Abiotic synthesis during the interaction of ferrous chloride–rich silicic fluids with marble under high-grade metamorphic conditions
Source: Proc Natl Acad Sci U S A. 2025 Aug 28;122(35):e2423043122. doi: 10.1073/pnas.2423043122 (PMC12415227; doi:10.1073/pnas.2423043122)
Supplement: Supplementary file 1 — Appendix 01 (PDF) [file pnas.2423043122.sapp.pdf]

PNAS

Supporting Information for

Abiotic synthesis during the interaction of ferrous chloride-rich silicic fluids with marble under high-grade metamorphic conditions

Chenhui Fei[a], Shun Guo[a]*, Yibing Li[b], Jingbo Liu[a, c]*

* Corresponding authors: Shun Guo and Jingbo Liu.
Email: guoshun@mail.iggcas.ac.cn (S. Guo); jingboliu@mail.iggcas.ac.cn (J.B. Liu)

This PDF file includes:

Supporting Text
Figures S1 to S5
Tables S1 to S7
SI References

Supporting Text

Estimates of fluid/rock ratios associated with metasomatism. The fluid/rock ratios for the metasomatic formation of the olivine marble and diopsidite were estimated on the basis of (1) the Fe^{2+} content in reactive fluids and (2) the amount of Fe^{2+} input per unit mass of marble (to form the olivine marble and diopsidite). The Fe^{2+} content in the reactive fluids can be determined by the solubility of Fe^{2+} under the metasomatic conditions (2.0 GPa and 780 °C; Fig. 3). Previous studies have demonstrated that the addition of Cl can increase the solubility of Fe^{2+} in fluids (refs. 1–3). The presence of Cl-bearing magnetite and halite grains indicates that the infiltrating fluids were Cl-saturated silicic solutions. The solubility of Fe^{2+} in H_2O–NaCl silicic fluids at 2 GPa and 800 °C is 0.095 mol/kg (ref. 4). The amounts of Fe^{2+} input into each 1 kg of dolomitic marble are 0.223–0.274 mol for the formation of olivine marble and 0.672–0.790 mol for the formation of diopsidite, respectively, on the basis of the results of the Ca or Mg conservation calculations (*SI Appendix*, Table S3). Therefore, using the solubility of Fe^{2+} (0.095 mol/kg), the minimum fluid/rock ratios required for the formation of olivine marble and diopsidite are estimated to be 2.3–2.9 and 7.0–8.4, respectively, assuming complete transfer of Fe from the fluid to the rock during metasomatism. In fact, these estimates represent the minimum fluid/rock ratios due to there exists the Fe partitioning between the fluid and the rock.

H_2 production calculations. The production of H_2 during the metasomatism of marbles occurs via the following general reaction:

$$2Fe^{2+} (aq) + 2H^{+} (aq) = 2Fe^{3+} (mineral) + H_2$$

Because the Fe in both the protolith (dolomitic marble) and the infiltrating fluids occurred as Fe^{2+}, all of the Fe^{3+} in the olivine marble and diopsidite should have formed through the reaction above. The molar ratio of Fe^{3+} to H_2 is 2:1 (i.e., a 1:1 molar ratio of Fe_2O_3 to H_2). Therefore, the amount of H_2 produced can be calculated via the Fe_2O_3 content in the rock using the following equation:

$$H_2{}^{produced}(mmol/kg) = \frac{m_{Fe_2O_3}}{M_{Fe}} \times 10 \times 1000$$

where $m_{Fe_2O_3}$ is the mass percentage (wt.%) of Fe_2O_3 in the rock (equivalent to the mass of Fe_2O_3 per 100 g of rock) and where M_{Fe} is the molar mass of Fe_2O_3 (159.69 g/mol). The content of Fe_2O_3 is then converted to mmol per kg of rock and then into moles, which is directly proportional to the moles of H_2 produced. In the olivine marble, the Fe_2O_3 content is 1.15 wt.% (*SI Appendix*, Table S2), corresponding to the production of 72 mmol H_2 during the formation of per kg of olivine marble. In the diopsidite, the Fe_2O_3 content is 2.26 wt.% (*SI Appendix*, Table S2), corresponding to the production of 142 mmol H_2 during the formation of per kg diopsidite.

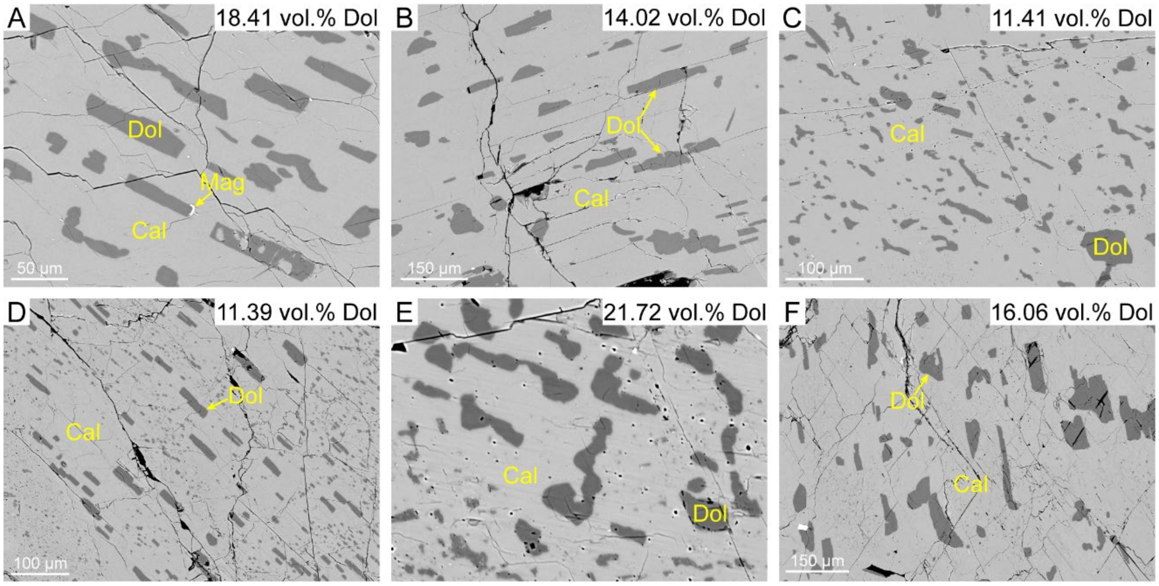

Fig. S1. Backscattered electron (BSE) images showing exsolved dolomite lamellae in calcite grains in olivine marble.

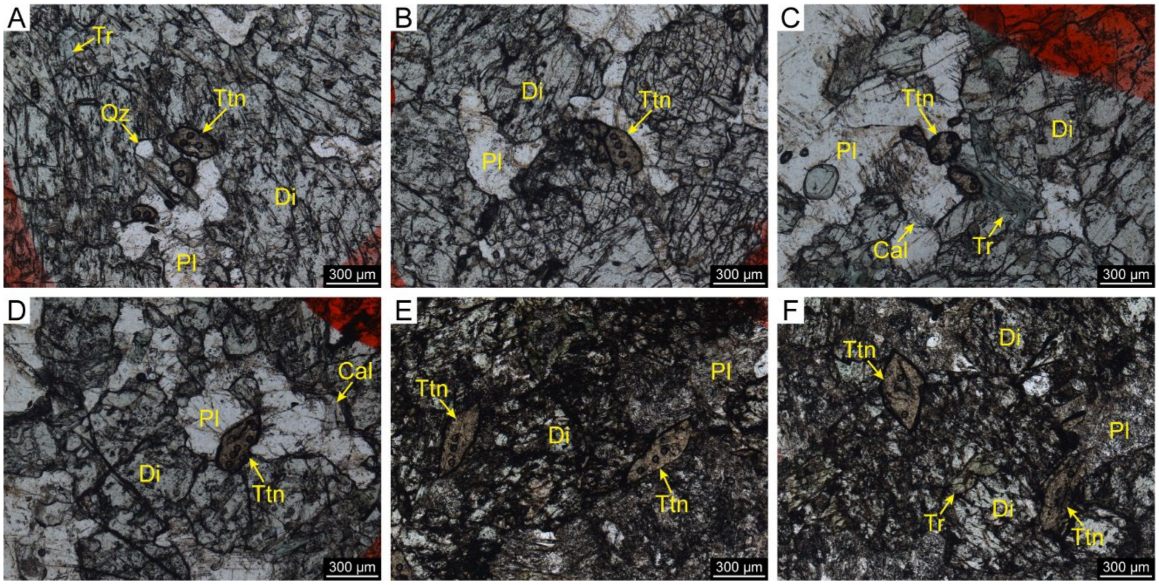

Fig. S2. Photomicrographs (plane-polarized light) showing titanite in diopsidite used for LA-ICP-MS analysis.

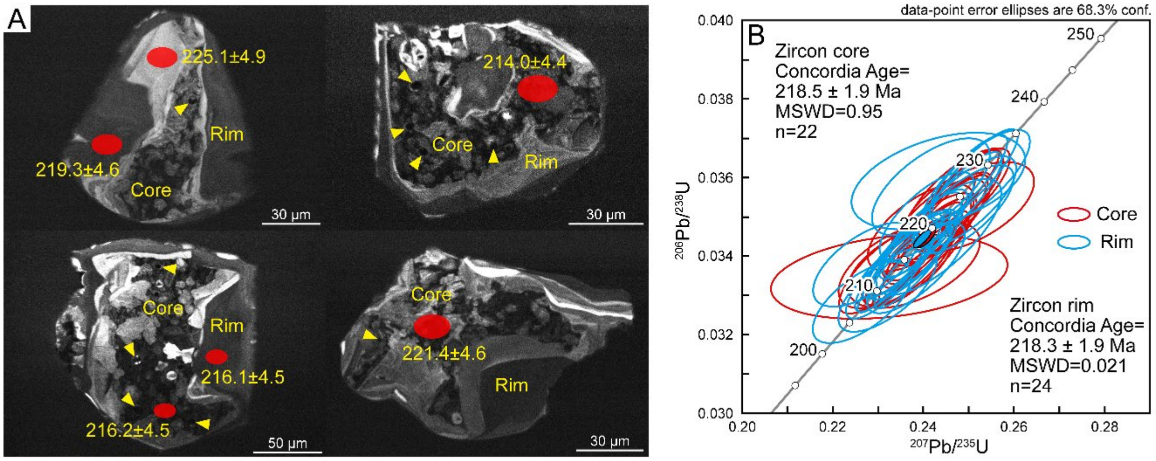

Fig. S3. *(A)* Representative CL images showing the core-rim structure of zircon from diopsidite. Inclusions mainly occur in the patchy core, marked by the yellow triangles. *(B)* U–Pb ages of zircons from diopsidite. The cores and rims give 218.5 ± 1.9 Ma and 218.3 ± 1.9 Ma Concordia ages, respectively.

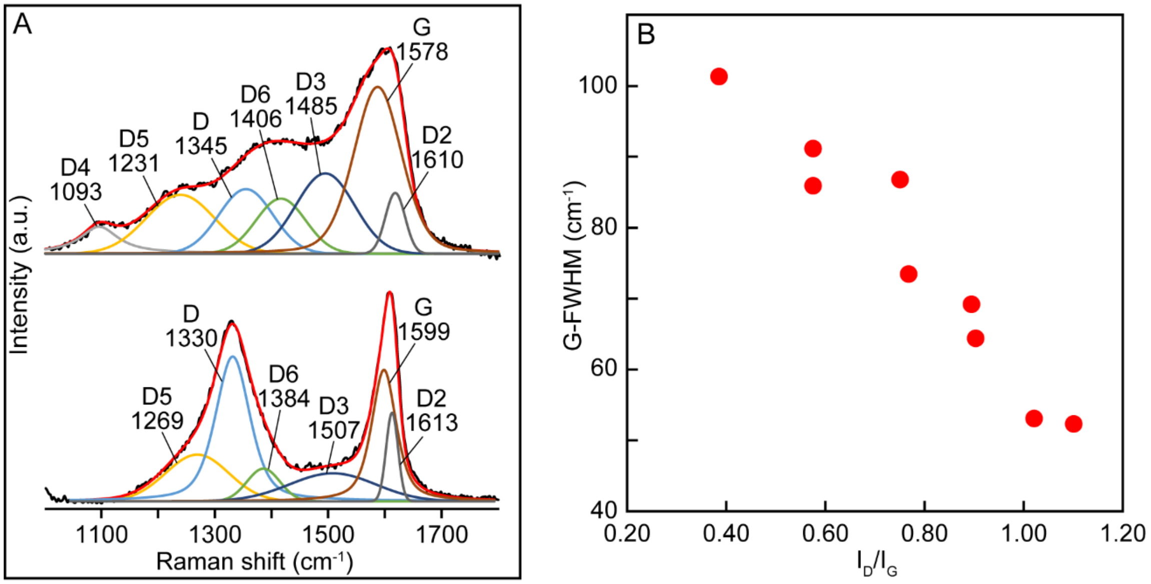

Fig. S4. *(A)* Raman spectra of DCM. Seven-bands fitting is shown, and the red-dish line is the fitting curve. *(B)* G-FWHN *vs.* I_D/I_G plot of DCM.

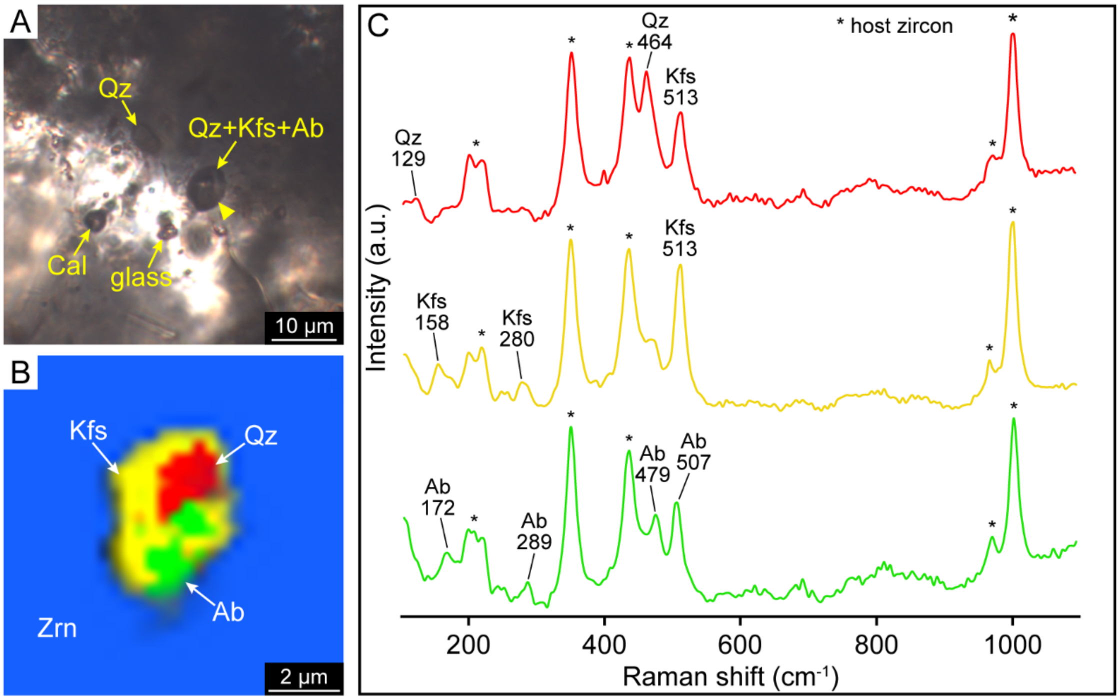

Fig. S5. *(A, B)* Photomicrograph (in plane-polarized light) and Raman map showing a melt inclusion in zircon from diopsidite, which is composed of Qz, Kfs, and Ab, indicating that zircon grew in the presence of melts. (C) Representative Raman spectra of the phases in the melt inclusion.

Table S1. Representative compositional analyses of minerals from dolomitic marble, olivine marble and diopsidite

Sample	18WH-5b			18WH-5c			
Rocks	dolomitic marble			Olivine marble			
Minerals	Dol	Dol	Dol	Dol	Dol	Cal	Cal
FeO	0.11	0.36	0.09	0.49	0.58	0.03	0.08
MnO	0.00	0.07	0.00	0.11	0.06	0.09	0.01
MgO	21.77	21.35	21.87	21.07	21.23	1.42	1.04
CaO	30.34	30.44	30.10	29.97	30.41	53.39	55.28
SrO	0.02	0.00	0.00	0.05	0.00	0.00	0.03
BaO	0.01	0.07	0.00	0.00	0.09	0.02	0.00
CO_2	47.69	47.48	47.55	46.98	47.51	43.67	44.69
Total	99.94	99.78	99.60	98.68	99.88	98.62	101.13
Fe^{2+}	0.003	0.009	0.002	0.013	0.015	0.001	0.002
Mn^{2+}	0.000	0.002	0.000	0.003	0.001	0.002	0.000
Mg^{2+}	0.997	0.982	1.004	0.980	0.976	0.071	0.051
Ca^{2+}	0.999	1.006	0.993	1.002	1.005	1.921	1.943
Sr^{2+}	0.000	0.000	0.000	0.001	0.000	0.000	0.001
Ba^{2+}	0.000	0.001	0.000	0.000	0.001	0.000	0.000
Fe/Fe+Mg	0.003	0.009	0.002	0.013	0.015	0.012	0.039

Table S1. (continued)

Sample	18WH-5c							18WH-2										
Rocks	Olivine marble							Diopsidite										
Minerals	Ol	Ol	Phl	Chu	Tr	Flr	Mag	Di	Di	Di	Tr	Phn	Bt	Ttn	Ep	Chl	Kfs	Pl
SiO_2	40.50	40.45	41.38	36.43	57.02	0.10	0.00	54.79	54.32	52.80	57.42	48.30	40.83	29.87	36.43	30.71	63.78	61.19
TiO_2	0.00	0.02	0.35	1.86	0.06	0.03	0.07	0.03	0.01	0.00	0.06	0.55	0.59	35.82	0.08	0.04	0.04	0.00
Al_2O_3	0.00	0.00	11.86	0.00	0.71	0.00	0.00	0.70	0.70	0.90	0.82	29.45	15.37	2.18	24.99	19.68	17.66	23.68
Cr_2O_3	0.00	0.00	0.00	0.00	0.03	0.00	0.05	0.06	0.03	0.00	0.00	0.00	0.03	0.00	0.01	0.00	0.05	0.02
FeO	4.15	4.22	2.74	3.25	1.83	0.01	94.92	2.79	4.80	6.30	3.12	2.74	6.46	0.54	9.24	6.14	0.08	0.10
MnO	0.15	0.16	0.07	0.11	0.06	0.00	0.04	0.19	0.28	0.20	0.14	0.00	0.16	0.04	0.10	0.22	0.00	0.00
NiO	0.03	0.00	0.00	0.00	0.02	0.04	0.03	0.04	0.02	0.00	0.02	0.02	0.00	0.00	0.00	0.01	0.01	0.02
MgO	56.00	54.44	26.84	54.91	23.64	1.95	0.10	17.25	15.67	15.00	22.60	3.43	21.00	0.01	0.10	30.18	0.04	0.00
CaO	0.02	0.05	0.11	0.01	13.27	70.86	0.03	24.48	24.17	24.70	13.06	0.02	0.06	27.50	24.09	0.05	0.00	5.15
Na_2O	0.00	0.01	0.16	0.00	0.21	0.25	0.05	0.14	0.28	0.40	0.10	0.11	0.03	0.03	0.00	0.00	0.10	8.66
K_2O	0.03	0.01	10.79	0.00	0.20	0.00	0.00	0.02	0.01	0.00	0.11	11.34	10.82	0.00	0.00	0.02	17.43	0.20
F	0.00	0.00	0.99	2.02	0.01	43.83	0.00	0.00	0.00	0.00	0.07	0.00	0.55	0.31	0.00	0.03	0.00	0.00
Cl	0.00	0.00	0.02	0.00	0.21	0.01	0.01	0.01	0.01	0.00	0.02	0.01	0.02	0.02	0.01	0.00	0.01	0.00
Total	100.88	99.35	94.89	98.58	97.27	99.12	95.29	100.50	100.29	100.30	97.49	95.96	95.68	96.18	95.04	87.05	99.19	99.00
O	4	4	22	17	23		32	6	6	6	23	22	22	20	12.5	28	8	8
Si	0.96	0.98	5.92	3.87	7.83		0.00	1.99	1.99	1.96	7.87	6.47	5.85	4.05	3.02	5.85	3.00	2.74
Al	0.00	0.00	2.00	0.00	0.12		0.00	0.00	0.03	0.04	0.13	4.65	2.60	0.35	2.44	4.43	0.98	1.25
Ti	0.00	0.00	0.04	0.15	0.01		0.02	0.03	0.00	0.00	0.01	0.06	0.06	3.65	0.01	0.00	0.00	0.00
Fe^{3+}	0.00	0.00	0.00	0.00	0.21		11.98	0.01	0.01	0.03	0.23	0.00	0.00	0.06	0.64	0.09	0.00	0.00
Mg	1.99	1.96	5.72	8.69	4.84		0.05	0.93	0.86	0.83	4.62	0.69	4.49	0.00	0.01	8.57	0.00	0.00
Fe^{2+}	0.08	0.09	0.33	0.29	0.00		14.92	0.06	0.10	0.11	0.13	0.31	0.77	0.00	0.00	0.89	0.00	0.00
Mn	0.00	0.00	0.01	0.01	0.01		0.01	0.01	0.01	0.01	0.02	0.00	0.02	0.01	0.01	0.04	0.00	0.00
Ca	0.00	0.00	0.02	0.00	1.95		0.00	0.95	0.95	0.98	1.92	0.00	0.01	4.00	2.14	0.01	0.00	0.25
Na	0.00	0.00	0.04	0.00	0.06		0.00	0.01	0.02	0.03	0.03	0.03	0.01	0.01	0.00	0.00	0.01	0.75
K	0.00	0.00	1.97	0.00	0.04		0.00	0.00	0.00	0.00	0.02	1.94	1.98	0.00	0.00	0.01	1.04	0.01
Fe/Fe+Mg	0.04	0.04	0.05	0.03	0.04		1.00	0.06	0.11	0.15	0.07	0.31	0.15	0.97	0.98	0.10		
								Wo=48.18<br>En=47.24<br>Fs=4.58	Wo=48.39<br>En=43.6<br>Fs=7.94	Wo=48.81<br>En=41.257<br>Fs=9.94							Ab=0.01<br>An=0.00<br>Or=0.99	Ab=0.74<br>An=0.24<br>Or=0.01

Table S2. Bulk compositions of dolomitic marble, olivine marble and diopsidite

**Rocks**	Dol-marble	Ol-marble	Diopsidite
		wt.%	
SiO_2	1.48	15.73	52.54
TiO_2	0.02	0.02	0.08
Al_2O_3	0.19	0.28	2.26
TFe_2O_3	0.25	2.06	4.28
Fe_2O_3[a]	0.00	1.15	2.26
FeO	0.23	0.82	1.82
MnO	0.03	0.10	0.18
MgO	22.95	19.39	14.98
CaO	29.93	30.25	22.82
Na_2O	0.00	0.00	0.57
K_2O	0.00	0.09	0.11
P_2O_5	0.02	0.01	0.00
LOI[b]	45.48	32.16	2.05
TOTAL	100.32	99.99	99.67

[a] Fe_2O_3 content was calculated using the total Fe and analysed FeO contents;
[b] LOI is predominantly from decarbonation of carbonate minerals.

Table S3. Mass balance calculations for the formation of olivine marble and diopsidite

Rocks	Dol-marble	Ol-marble	Diopsidite	Gain or loss [b] in Ol-marble			Gain or loss in diopsidite		
	Concentration (mol/kg)			Ca conservation	Mg conservation	Fe conservation	Ca conservation	Mg conservation	Fe conservation
SiO_2	0.246	2.618	8.744	90.49	92.05	24.13	97.85	98.16	52.80
TiO_2	0.002	0.002	0.010	-1.06	15.51	-87.60	81.43	84.10	-75.50
Al_2O_3	0.019	0.027	0.222	31.42	42.67	-81.73	93.59	94.51	-29.01
TFeO [a]	0.032	0.258	0.536	87.47	89.52	0.00	95.45	96.10	0.00
MnO	0.004	0.014	0.026	73.68	78.00	-52.38	89.64	91.13	-56.07
MgO	5.695	4.811	3.717	-16.41	0.00	-89.52	-14.39	0.00	-96.10
CaO	5.337	5.394	4.069	0.00	16.41	-87.47	0.00	14.39	-95.45
Na_2O	0.000	0.000	0.092	-	-	-	100.00	100.00	100.00
K_2O	0.000	0.009	0.012	95.35	96.12	62.92	97.32	97.71	41.21
P_2O_5	0.001	0.001	0.000	-40.13	-28.38	-92.50	-61.57	-55.11	-98.25
CO_2	10.334	7.307	0.466	-30.04	-16.30	-91.23	-94.09	-93.09	-99.73

[a] Iron was calculated as TFeO;

[b] Gain of a component = ($C_{i \text{ in Ol-marble or diopsidite}}$ × $C_{m \text{ in Dol-marble}}$/$C_{m \text{ in Ol-marble or diopsidite}}$ - $C_{i \text{ in Dol-marble}}$)/($C_{i \text{ in Ol marble or diopsidite}}$ × $C_{m \text{ in Dol-marble}}$/$C_{m \text{ in Ol-marble or diopsidite}}$) × 100; Loss of a component = ($C_{i \text{ in Ol-marble or diopsidite}}$ × $C_{m \text{ in Dol-marble}}$/$C_{m \text{ in Ol-marble or diopsidite}}$ - $C_{i \text{ in Dol-marble}}$)/$C_{i \text{ in Dol-marble}}$ × 100, in which C_i is the concentration of component *i*, and C_m is the concentration of a conserved component (CaO, MgO, or FeO).

Table S4. Metamorphic temperature estimated using the calcite-dolomite geothermometer for the formation of olivine marble

Sample	Cal vol. %	Dol vol. %	Dol/(Dol+Cal) (mol)	Calculated calcite formula before dolomite exsolution	T(°C)
18WH-2a-1	81.59	18.41	0.205	$(Ca_{0.870}Mg_{0.126}Fe_{0.004})CO_3$	731
18WH-2a-2	89.15	10.85	0.122	$(Ca_{0.891}Mg_{0.102}Fe_{0.007})CO_3$	678
18WH-2a-3	88.61	11.39	0.128	$(Ca_{0.889}Mg_{0.104}Fe_{0.007})CO_3$	682
18WH-2a-4	85.98	14.02	0.157	$(Ca_{0.870}Mg_{0.123}Fe_{0.007})CO_3$	724
18WH-2a-5	83.94	16.06	0.179	$(Ca_{0.847}Mg_{0.135}Fe_{0.008})CO_3$	747
18WH-5c-1	88.59	11.41	0.128	$(Ca_{0.888}Mg_{0.111}Fe_{0.001})CO_3$	699
18WH-5c-2	80.80	19.20	0.214	$(Ca_{0.851}Mg_{0.148}Fe_{0.001})CO_3$	773
18WH-5c-3	78.28	21.72	0.241	$(Ca_{0.839}Mg_{0.160}Fe_{0.001})CO_3$	794

The densities for the calculation of calcite compositions before dolomite exsolution are 2.72 g/cm^3 for calcite and 2.86 g/cm^3 for dolomite. Calcite-Dolomite geothermometer from ref. 5.

Table S5. Metamorphic temperature estimates using the Zr-in-titanite thermobarometer for the formation of diopsidite

No.	Zr (sphene, ppm)	P (GPa)	T (°C)	P (GPa)	T (°C)	P (GPa)	T (°C)	P (GPa)	T (°C)	P (GPa)	T (°C)	P (GPa)	T (°C)	P (GPa)	T (°C)
18WH3a - 1	441	2.0	950	1.6	901	1.2	852	1.0	828	0.8	803	0.4	755	0.2	730
18WH3a - 2	368	2.0	937	1.6	889	1.2	841	1.0	817	0.8	793	0.4	744	0.2	720
18WH3a - 3	317	2.0	928	1.6	880	1.2	832	1.0	808	0.8	784	0.4	736	0.2	712
18WH3a - 4	453	2.0	951	1.6	902	1.2	854	1.0	829	0.8	805	0.4	756	0.2	732
18WH3a - 5	378	2.0	939	1.6	891	1.2	842	1.0	818	0.8	794	0.4	746	0.2	722
18WH3a - 6	344	2.0	933	1.6	885	1.2	837	1.0	813	0.8	789	0.4	741	0.2	717
18WH3a - 7	318	2.0	928	1.6	880	1.2	832	1.0	808	0.8	784	0.4	736	0.2	712
18WH3a - 8	313	2.0	927	1.6	879	1.2	831	1.0	807	0.8	783	0.4	735	0.2	712
18WH3a - 9	398	2.0	943	1.6	894	1.2	846	1.0	821	0.8	797	0.4	749	0.2	724
18WH3a - 10	363	2.0	937	1.6	888	1.2	840	1.0	816	0.8	792	0.4	744	0.2	719
18WH3a - 11	386	2.0	941	1.6	892	1.2	844	1.0	820	0.8	795	0.4	747	0.2	723
18WH3a - 12	401	2.0	943	1.6	895	1.2	846	1.0	822	0.8	798	0.4	749	0.2	725
18WH3a - 13	338	2.0	932	1.6	884	1.2	836	1.0	812	0.8	788	0.4	740	0.2	716
18WH3a - 14	302	2.0	924	1.6	877	1.2	829	1.0	805	0.8	781	0.4	733	0.2	710
18WH3a - 15	423	2.0	947	1.6	898	1.2	849	1.0	825	0.8	801	0.4	752	0.2	728
18WH3a - 16	339	2.0	932	1.6	884	1.2	836	1.0	812	0.8	788	0.4	740	0.2	716
18WH3a - 17	503	2.0	958	1.6	909	1.2	860	1.0	836	0.8	811	0.4	762	0.2	737
18WH3a - 18	471	2.0	954	1.6	905	1.2	856	1.0	832	0.8	807	0.4	758	0.2	734
18WH3b - 1	187	2.0	894	1.6	848	1.2	801	1.0	778	0.8	754	0.4	708	0.2	685
18WH3b - 2	280	2.0	920	1.6	872	1.2	824	1.0	801	0.8	777	0.4	729	0.2	706
18WH3b - 3	188	2.0	894	1.6	848	1.2	801	1.0	778	0.8	755	0.4	708	0.2	685
18WH3b - 4	264	2.0	916	1.6	868	1.2	821	1.0	797	0.8	774	0.4	726	0.2	702
18WH3b - 5	190	2.0	895	1.6	849	1.2	802	1.0	779	0.8	755	0.4	709	0.2	686
18WH3b - 6	172	2.0	889	1.6	843	1.2	796	1.0	773	0.8	750	0.4	704	0.2	681
18WH3b - 7	209	2.0	901	1.6	854	1.2	807	1.0	784	0.8	761	0.4	714	0.2	690
18WH3b - 8	168	2.0	888	1.6	841	1.2	795	1.0	772	0.8	749	0.4	703	0.2	679
18WH3b - 9	251	2.0	913	1.6	865	1.2	818	1.0	795	0.8	771	0.4	724	0.2	700
18WH3b - 10	169	2.0	888	1.6	842	1.2	795	1.0	772	0.8	749	0.4	703	0.2	680
18WH3b - 11	316	2.0	927	1.6	880	1.2	832	1.0	808	0.8	784	0.4	736	0.2	712
18WH3b - 14	164	2.0	886	1.6	840	1.2	794	1.0	771	0.8	748	0.4	701	0.2	678
18WH3b - 15	235	2.0	909	1.6	861	1.2	814	1.0	791	0.8	767	0.4	720	0.2	696
18WH3b - 16	219	2.0	904	1.6	857	1.2	810	1.0	787	0.8	763	0.4	716	0.2	693
18WH3b - 17	326	2.0	929	1.6	881	1.2	834	1.0	810	0.8	786	0.4	738	0.2	714
18WH3b - 18	273	2.0	918	1.6	871	1.2	823	1.0	799	0.8	776	0.4	728	0.2	704
18WH3b - 19	229	2.0	907	1.6	860	1.2	813	1.0	789	0.8	766	0.4	719	0.2	695

18WH3b - 20	240	2.0	910	1.6	863	1.2	815	1.0	792	0.8	768	0.4	721	0.2	698
18WH3b - 21	204	2.0	900	1.6	853	1.2	806	1.0	783	0.8	759	0.4	713	0.2	689
18WH3b - 22	177	2.0	891	1.6	845	1.2	798	1.0	775	0.8	752	0.4	705	0.2	682
18WH3b - 23	310	2.0	926	1.6	878	1.2	830	1.0	807	0.8	783	0.4	735	0.2	711
18WH3b - 24	254	2.0	913	1.6	866	1.2	819	1.0	795	0.8	771	0.4	724	0.2	700
18WH3b - 25	169	2.0	888	1.6	842	1.2	795	1.0	772	0.8	749	0.4	703	0.2	680
18WH3b - 26	185	2.0	894	1.6	847	1.2	801	1.0	777	0.8	754	0.4	708	0.2	684
18WH2b - 1	337	2.0	932	1.6	884	1.2	836	1.0	811	0.8	787	0.4	739	0.2	715
18WH2b - 2	550	2.0	965	1.6	915	1.2	866	1.0	841	0.8	816	0.4	767	0.2	742
18WH2b - 4	337	2.0	932	1.6	884	1.2	836	1.0	811	0.8	787	0.4	739	0.2	715
18WH2b - 5	323	2.0	929	1.6	881	1.2	833	1.0	809	0.8	785	0.4	737	0.2	713
18WH2b - 6	343	2.0	933	1.6	885	1.2	837	1.0	813	0.8	789	0.4	740	0.2	716
18WH2b - 7	319	2.0	928	1.6	880	1.2	832	1.0	808	0.8	784	0.4	736	0.2	712
18WH2b - 8	406	2.0	944	1.6	895	1.2	847	1.0	823	0.8	798	0.4	750	0.2	726
18WH2b - 9	356	2.0	935	1.6	887	1.2	839	1.0	815	0.8	791	0.4	742	0.2	718
18WH2b - 10	341	2.0	932	1.6	884	1.2	836	1.0	812	0.8	788	0.4	740	0.2	716

Table S6. SIMS U–Pb analytical results of zircons

Sample/ spot #	[U] ppm	[Th] ppm	[Pb] ppm	Th/U meas	$^{207}Pb/^{235}U$	±σ %	$^{206}Pb/^{238}U$	±σ %	r	Disc. % conv.	$^{207}Pb/^{206}Pb$	±σ	$^{207}Pb/^{235}U$	±σ	$^{206}Pb/^{238}U$	±σ	207-corr age (Ma)	±σ	f_{206}%
Core																			
17WH-3@1	2534	1846	112	0.728	0.24121	2.55	0.0344	2.09	0.82052	-7.6	235.6	33.7	219.4	5.0	217.9	4.5	217.8	4.5	* 0.11
17WH-3@11	2186	1400	97	0.641	0.24679	2.89	0.0355	2.21	0.76368	5.8	212.9	43.2	224.0	5.8	225.0	4.9	225.1	4.9	0.90
17WH-3@15	2649	1303	111	0.492	0.24314	2.90	0.0349	2.10	0.72379	-0.3	221.6	46.3	221.0	5.8	220.9	4.6	220.9	4.6	0.45
17WH-3@16	2044	675	84	0.330	0.24787	2.36	0.0356	2.08	0.88228	4.3	216.5	25.8	224.8	4.8	225.6	4.6	225.7	4.7	{0.01}
17WH-3@18	1627	207	60	0.127	0.24012	2.72	0.0340	2.32	0.85083	-15.2	253.1	32.9	218.5	5.4	215.3	4.9	215.1	4.9	* 0.00
17WH-3@20	5822	1537	223	0.264	0.23673	2.22	0.0338	2.10	0.94536	-8.3	233.2	16.7	215.7	4.3	214.1	4.4	214.0	4.4	{0.19}
17WH-3@22	5345	721	194	0.135	0.23681	2.40	0.0341	2.10	0.87493	1.9	212.1	27.0	215.8	4.7	216.1	4.5	216.2	4.5	0.58
17WH-3@24	1045	488	43	0.467	0.24136	2.86	0.0345	2.12	0.74187	-4.7	229.3	44.2	219.5	5.7	218.6	4.6	218.6	4.6	* 0.13
17WH-3@25	1501	1756	55	1.169	0.23249	7.40	0.0335	2.11	0.28459	0.1	212.0	165.0	212.3	14.3	212.3	4.4	212.2	4.5	3.06
17WH-3@26	2516	845	101	0.336	0.23947	3.23	0.0347	2.11	0.65468	12.8	195.4	56.7	218.0	6.4	220.1	4.6	220.2	4.6	0.63
17WH-3@30	1867	905	71	0.484	0.23796	4.03	0.0338	2.15	0.53307	-12.7	244.7	78.6	216.7	7.9	214.2	4.5	214.0	4.5	1.62
17WH-3@31	2546	1802	122	0.708	0.23104	2.75	0.0336	2.11	0.76933	13.7	187.8	40.8	211.1	5.2	213.1	4.4	213.3	4.5	0.60
17WH-3@44	4716	7030	249	1.491	0.24284	2.33	0.0349	2.13	0.91355	1.9	217.0	22.0	220.7	4.6	221.1	4.6	221.1	4.7	0.18
17WH-3@47	2737	2758	124	1.008	0.23668	2.85	0.0338	2.10	0.73655	-6.6	229.2	44.5	215.7	5.6	214.5	4.4	214.4	4.5	0.83
17WH-3@48	5953	1213	226	0.204	0.23579	2.40	0.0338	2.10	0.87669	-2.6	220.0	26.7	215.0	4.7	214.5	4.4	214.5	4.5	0.67
17WH-3@49	2754	2562	126	0.930	0.24001	2.42	0.0343	2.10	0.86787	-5.8	230.5	27.7	218.4	4.8	217.3	4.5	217.2	4.5	0.14
17WH-3@52	2293	381	88	0.166	0.24539	2.62	0.0350	2.09	0.79536	-4.5	232.2	36.7	222.8	5.3	221.9	4.6	221.9	4.6	* 0.00
17WH-3@57	2511	336	91	0.134	0.23382	3.53	0.0337	2.09	0.59393	3.5	206.8	65.8	213.3	6.8	213.9	4.4	214.0	4.4	1.15
17WH-3@58	2122	913	88	0.430	0.24530	2.59	0.0350	2.09	0.80450	-6.4	236.2	35.6	222.8	5.2	221.5	4.5	221.4	4.6	0.31
17WH-3@59	2512	1387	107	0.552	0.24507	2.47	0.0350	2.12	0.86010	-5.5	234.2	29.0	222.6	4.9	221.5	4.6	221.4	4.6	0.22
17WH-3@60	977	63	37	0.065	0.24890	2.70	0.0356	2.15	0.79720	-1.5	228.7	37.6	225.7	5.5	225.4	4.8	225.4	4.8	* 0.03
17WH-3@63	1204	306	48	0.254	0.25004	3.79	0.0353	2.14	0.56419	-14.5	260.6	71.9	226.6	7.7	223.3	4.7	223.1	4.7	* 0.00
Rim																			
17WH-3@05	2447	164	86	0.067	0.23212	3.08	0.0332	2.14	0.69344	-9.0	230.7	51.3	211.9	5.9	210.3	4.4	210.1	4.4	1.36
17WH-3@06	1295	30	49	0.023	0.24756	2.60	0.0355	2.21	0.84982	0.4	223.9	31.6	224.6	5.2	224.7	4.9	224.7	4.9	* 0.00
17WH-3@10	1928	11	70	0.006	0.24039	2.56	0.0346	2.12	0.82767	2.7	213.5	33.2	218.7	5.0	219.2	4.6	219.3	4.6	* 0.07
17WH-3@13	2488	16	88	0.007	0.23367	2.46	0.0337	2.12	0.85944	1.5	210.4	29.2	213.2	4.7	213.5	4.4	213.5	4.5	0.14
17WH-3@14	1376	13	[50]	0.009	0.24072	2.84	0.0350	2.11	0.74372	18.4	188.0	44.2	219.0	5.6	221.9	4.6	222.1	4.7	* 0.16
17WH-3@17	929	3	[32]	0.003	0.23839	2.98	0.0337	2.25	0.75684	-16.3	254.4	44.8	217.1	5.8	213.7	4.7	213.4	4.8	* 0.16
17WH-3@19	2305	9	82	0.004	0.23776	2.36	0.0337	2.09	0.88331	-13.1	245.5	25.5	216.6	4.6	213.9	4.4	213.7	4.4	* 0.04
17WH-3@21	1794	30	65	0.017	0.23710	2.43	0.0341	2.09	0.86168	0.4	215.3	28.5	216.0	4.7	216.1	4.4	216.1	4.5	* 0.02
17WH-3@23	3809	37	138	0.010	0.23937	2.53	0.0345	2.10	0.83034	5.1	208.4	32.7	217.9	5.0	218.8	4.5	218.8	4.6	0.15
17WH-3@27	951	28	35	0.029	0.24337	3.74	0.0345	2.82	0.75442	-12.3	248.7	56.6	221.2	7.5	218.6	6.1	218.4	6.1	* 0.00

17WH-3@29	638	34	24	0.053	0.24167	3.54	0.0358	2.28	0.64211	54.0	148.0	63.7	219.8	7.0	226.5	5.1	227.0	5.1	{0.09}
17WH-3@33	939	17	34	0.018	0.23755	2.80	0.0340	2.21	0.79040	-3.2	222.9	39.7	216.4	5.5	215.8	4.7	215.8	4.7	* 0.00
17WH-3@34	2882	64	100	0.022	0.22650	3.17	0.0328	2.08	0.65720	5.9	196.8	55.6	207.3	6.0	208.2	4.3	208.3	4.3	* 0.19
17WH-3@35	1139	9	40	0.008	0.23245	2.70	0.0335	2.21	0.82108	2.6	207.4	35.7	212.2	5.2	212.7	4.6	212.7	4.7	{0.03}
17WH-3@36	1201	124	46	0.104	0.24884	2.93	0.0352	2.12	0.72261	-12.3	253.6	46.5	225.6	5.9	223.0	4.6	222.8	4.7	* 0.01
17WH-3@37	2868	78	102	0.027	0.23372	2.32	0.0336	2.11	0.90843	0.4	212.4	22.5	213.3	4.5	213.3	4.4	213.3	4.5	{0.02}
17WH-3@39	775	38	29	0.049	0.24330	5.19	0.0359	2.26	0.43470	48.3	154.2	109.6	221.1	10.4	227.5	5.0	227.9	5.1	* 0.38
17WH-3@41	2614	16	94	0.006	0.23743	2.64	0.0340	2.18	0.82330	-4.0	224.3	34.7	216.3	5.2	215.6	4.6	215.5	4.6	* 0.04
17WH-3@43	1036	37	39	0.035	0.24950	2.90	0.0354	2.13	0.73588	-9.1	246.3	45.2	226.2	5.9	224.2	4.7	224.1	4.7	* 0.25
17WH-3@45	1365	79	52	0.058	0.24905	2.51	0.0359	2.13	0.85115	9.6	208.0	30.5	225.8	5.1	227.5	4.8	227.6	4.8	{0.00}
17WH-3@51	1429	60	55	0.042	0.25141	2.47	0.0361	2.10	0.85019	4.2	219.5	30.1	227.7	5.1	228.5	4.7	228.6	4.8	{0.09}
17WH-3@53	666	23	25	0.035	0.25243	2.89	0.0360	2.18	0.75293	-2.0	232.6	44.0	228.5	5.9	228.1	4.9	228.1	4.9	* 0.02
17WH-3@61	2119	24	76	0.011	0.23539	2.47	0.0340	2.09	0.84475	5.9	203.8	30.7	214.6	4.8	215.6	4.4	215.7	4.5	0.23
17WH-3@62	2290	26	[82]	0.012	0.22728	3.36	0.0338	2.09	0.62050	56.9	137.4	62.0	208.0	6.3	214.2	4.4	214.7	4.4	0.69

Table S7. Band-fitting parameters of DCM in inclusions

No.		L-1	L-3	M-1	M-3	M-7	H-1	H-2	H-3-1	H-4
D	shift	1349.0	1345.0	1343.0	1342.0	1334.0	1330.0	1336.0	1338.0	1328.0
	FWHM	92.2	110.8	77.0	89.5	88.8	71.0	78.6	83.2	71.2
	Amp	752.8	2524.9	626.3	984.3	440.5	673.3	1815.6	5849.1	1816.3
	Area	73890.0	297700.0	51340.0	93770.0	41630.0	62310.0	186300.0	517900.0	191200.0
D2	shift	1616.0	1610.0	1610.0	1602.0	1610.0	1613.0	1611.0	1611.0	1610.0
	FWHM	40.0	41.8	44.8	37.5	36.6	25.2	32.7	29.4	24.0
	Amp	274.2	2376.4	694.1	666.5	145.3	412.1	1284.6	2913.8	983.1
	Area	17240.0	105800.0	39180.0	26590.0	8363.9	11080.0	44650.0	110200.0	25160.0
D3	shift	1500.0	1485.0	1476.0	1515.0	1487.0	1507.0	1506.0	1481.0	1506.0
	FWHM	110.7	122.3	136.1	170.5	192.0	180.8	155.5	206.4	182.0
	Amp	591.4	3146.1	431.2	732.2	229.0	129.2	565.5	2281.7	380.1
	Area	69660.0	409700.0	65640.0	174600.0	46800.0	24870.0	93600.0	501300.0	73640.0
D4	shift	-	1093.0	-	-	-	-	-	-	-
	FWHM	-	77.8	-	-	-	-	-	-	-
	Amp	-	2524.9	-	-	-	-	-	-	-
	Area	-	128600.0	-	-	-	-	-	-	-
D5	shift	1270.0	1232.0	1264.0	1275.0	1253.0	1269.0	1281.0	1267.0	1267.0
	FWHM	198.4	138.3	142.0	154.0	130.4	128.7	145.6	85.7	150.1
	Amp	485.2	2309.9	491.1	751.2	230.0	216.4	928.0	2908.6	625.6
	Area	132200.0	340000.0	74220.0	123100.0	37030.0	29640.0	157000.0	296800.0	99960.0
D6	shift	1428.0	1406.0	1395.0	1403.0	1387.0	1384.0	1387.0	1393.0	1380.0
	FWHM	55.1	102.7	98.5	112.4	90.4	68.0	96.3	86.1	68.6
	Amp	454.6	2160.5	548.0	641.9	216.1	151.9	925.2	1980.0	368.6
	Area	39320.0	236300.0	84500.0	90100.0	25100.0	11000.0	137900.0	199200.0	26910.0
G	shift	1581.0	1578.0	1591.0	1597.0	1597.0	1599.0	1596.0	1597.0	1599.0
	FWHM	91.1	101.3	85.9	86.7	73.4	52.3	69.2	64.4	53.1
	Amp	1308.3	6549.0	1088.4	1311.0	573.6	611.7	2028.9	6476.8	1778.5
	Area	138700.0	782900.0	116900.0	128200.0	58370.0	43810.0	171900.0	491600.0	123600.0
I(D)/I(G)		0.58	0.39	0.58	0.75	0.77	1.10	0.89	0.90	1.02
H:C		0.69	0.28	0.36	0.64	0.43	0.42	0.58	0.38	0.53

SI References

1. I. M. Chou, H. P. Eugster, Solubility of magnetite in supercritical chloride solutions. *Am. J. Sci.* **277**, 1296–1314 (1977).

2. N. Z. Boctor, R. K. Popp, J. D. Frantz, Mineral-solution equilibria—IV. Solubilities and the thermodynamic properties of $FeCl_2^0$ in the system Fe_2O_3–H_2–H_2O–HCl. *Geochim. Cosmochim. Acta* **44**, 1509–1518 (1980).

3. L. Scholten et al., Solubility and speciation of iron in hydrothermal fluids. *Geochim. Cosmochim. Acta* **252**, 126–143 (2019).

4. C. Tiraboschi et al., Preferential mobilisation of oxidized iron by slab-derived hydrous silicate melts. *Geochem. Perspect. Lett.* **24**, 43–47 (2023).

5. L. M. Anovitz, E. J. Essene, Phase equilibria in the system $CaCO_3$−$MgCO_3$−$FeCO_3$. *J. Petrol*. **28**, 389–414 (1987).
